# Acetylene and Ethylene Adsorption during Floating Fe Catalyst Formation at the Onset of Carbon Nanotube Growth and the Effect of Sulfur Poisoning: a DFT Study

**DOI:** 10.1021/acs.inorgchem.4c01830

**Published:** 2024-07-10

**Authors:** Balázs Orbán, Tibor Höltzl

**Affiliations:** † Department of Inorganic and Analytical Chemistry, Budapest University of Technology and Economics, Műegyetem rkp. 3., H-1111 Budapest, Hungary; ‡ HUN-REN-BME Computation Driven Research Group, Műegyetem rkp. 3., H-1111 Budapest, Hungary; § Furukawa Electric Institute of Technology, Késmárk utca 28/A, H-1158 Budapest, Hungary

## Abstract

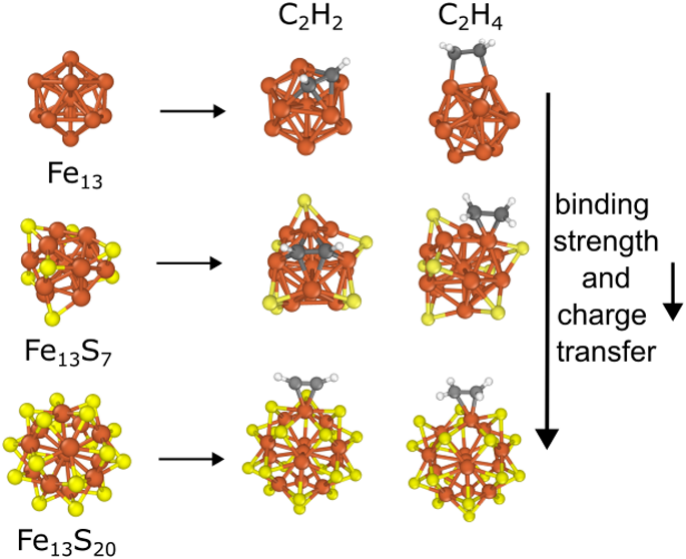

Here, we investigated the adsorption of acetylene and ethylene on iron clusters and nanoparticles, which is a crucial aspect in the nascent phase of carbon nanotube growth by floating catalyst chemical vapor deposition (FCCVD). The effect of sulfur on adsorption was also studied due to its indispensable role in the process and its commonly known impact on metal catalyst poisoning. We performed systematic density functional theory (DFT) computations, considering numerous adsorption configurations and iron particles of various sizes (Fe*_n_*, *n* = 3–10, 13, 55). We found that acetylene binds significantly more strongly than ethylene and prefers different adsorption sites. The presence of sulfur decreased the adsorption strength only in the immediate proximity of the adsorbate, suggesting that the effect of sulfur is mainly of steric origin while electronic effects play only a minor role. Higher sulfur coverage of the catalyst surface significantly weakened the binding of acetylene or ethylene. To further investigate this interaction, Bader’s atoms in molecules (AIM) analysis and charge density difference (CDD) were used, which showed electron transfer from iron clusters or nanoparticles to the adsorbate molecules. The charge transfer exhibited a decreasing trend as sulfur coverage increased. These results can also contribute to the understanding of other iron-based catalytic processes involving hydrocarbons and sulfur, such as the Fischer–Tropsch synthesis.

## Abstract 2

In this study, we explore acetylene and ethylene adsorption on iron clusters and nanoparticles, which are crucial in heterogeneous catalysis processes like floating catalyst chemical vapor deposition and the Fischer−Tropsch synthesis. The impact of sulfur on adsorption is also examined due to its indispensable role in both methods. Using density functional theory, we investigate numerous adsorption configurations. Our findings, supported by electronic structure analyses, enhance the understanding of iron-catalyzed hydrocarbon reactions and the poisoning effect of sulfur.

## 1 Introduction

In the realm of nanomaterials research, carbon nanotubes (CNTs) have emerged as a captivating subject of study since their preparation in 1991.^1^ Due to their outstanding mechanical, electrical, and thermal properties, various potential applications have been found in the fields of electronics, optoelectronics, composites, and energy storage.^2−5^ Thus, the controlled growth of CNTs, in terms of both structure and alignment, has become a focal point for researchers seeking to harness the extraordinary attributes of these materials for numerous technological advancements. Out of the several synthesis techniques developed over the past few decades, catalytic chemical vapor deposition (CCVD), also employed in the first synthesis of CNTs,^6^ stands out as a promising candidate due to its high controllability, coupled with the potential for scalable production.^7−9^ In CCVD, the growth of CNTs occurs on the surface of the transition metal catalyst nanoparticles from the dissociation of carbon-containing gas at an elevated temperature. Although several method variations exist, one of them involves the in situ formation of catalyst nanoparticles within the gas flow. This technique is known as floating catalyst chemical vapor deposition (FCCVD).^10−18^

**Scheme 1:**
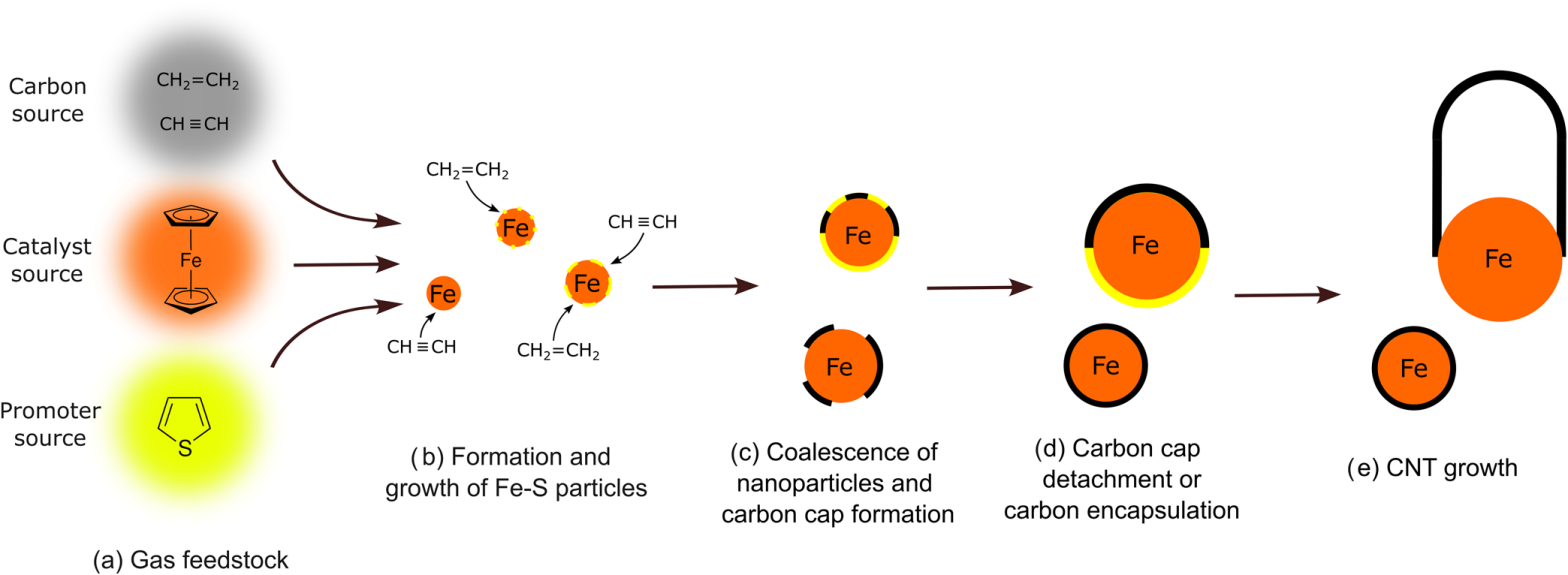
Schematic Representation of CNT Growth in the FCCVD Method

Typically, ferrocene is used as the iron source (Scheme 1a). Initially, ferrocene begins to thermally decompose at around 500 °C, leading to the formation of the first nuclei of iron nanoparticles.^19^ These small iron clusters catalyze the decomposition of further ferrocene, which facilitates continued growth.^20^ Previous molecular dynamics simulations indicated that the growing iron clusters remain free of hydrocarbon species at this early stage of the process when hydrogen, a common carrier gas, is present.^21^ The evolution of structure and energetic properties of iron clusters have also been extensively studied using density functional theory (DFT) computations.^22−25^ It was found that Fe*_n_* (*n* = 2–15) clusters exhibit close-packed structures, such as tetrahedral binding for smaller and icosahedral motif for larger number of iron atoms. Among them, Fe_13_ is outstandingly stable due to the completion of an icosahedral shell, which has also been observed experimentally.^26^

Simultaneously with the growth of iron clusters and nanoparticles in the FCCVD of CNT, the adsorption and dissociation of the carbon source occur on the catalyst surface (Scheme 1b).^27^ Many different carbon sources have been used successfully in the FCCVD technique; the most common are hydrocarbons such as methane,^15^ ethylene or acetylene,^12,28^ but alcohols have also shown to be promising.^11,16^ The growth of CNT on metal nanoparticles has been widely studied using molecular dynamics-based computational methods.^29−47^ The products of the dissociation are carbon atoms and dimers dissolved in iron particles, which lead to the formation of the initial CNT structure (carbon cap) on the nanoparticle surface (Scheme 1c,d) and the subsequent growth of CNT (Scheme 1e) on oversaturation. A byproduct of both carbon source and ferrocene decomposition is the hydrogen atoms adsorbed on the catalyst surface. However, it has been shown that they do not passivate the surface, as they can recombine with other hydrogen adatoms and desorb as molecular hydrogen.^21^ Moreover, the presence of hydrogen can contribute to preventing catalyst deactivation and even help the Cp rings from ferrocene to further decompose to smaller hydrocarbon molecules (such as acetylene and ethylene), providing more carbon sources for CNT growth. Because iron catalysts are also used in several other heterogeneous catalytic processes, for instance, the Fischer–Tropsch synthesis for hydrocarbon conversion,^48−52^ several studies reported the adsorption and dissociation of small molecules such as H_2_, CO, CH_3_OH, or CH_4_ on iron clusters of different sizes.^53−55^ It was found that during adsorption, the H–H, C–O, and C–H bond lengths increase, while their stretching vibrational modes are red-shifted, indicating the catalytic activation due to the interaction with the iron cluster.^53,54^ The adsorption strength of CH_4_ on Fe*_n_* shows strong size dependency only for *n* ≤ 6, as the adsorption energy fluctuates in the range of −0.5 to −0.1 eV, while the adsorption exhibits smaller size dependency for larger clusters, ranging between −0.15 and −0.3 eV.^55^

**Scheme 2:**
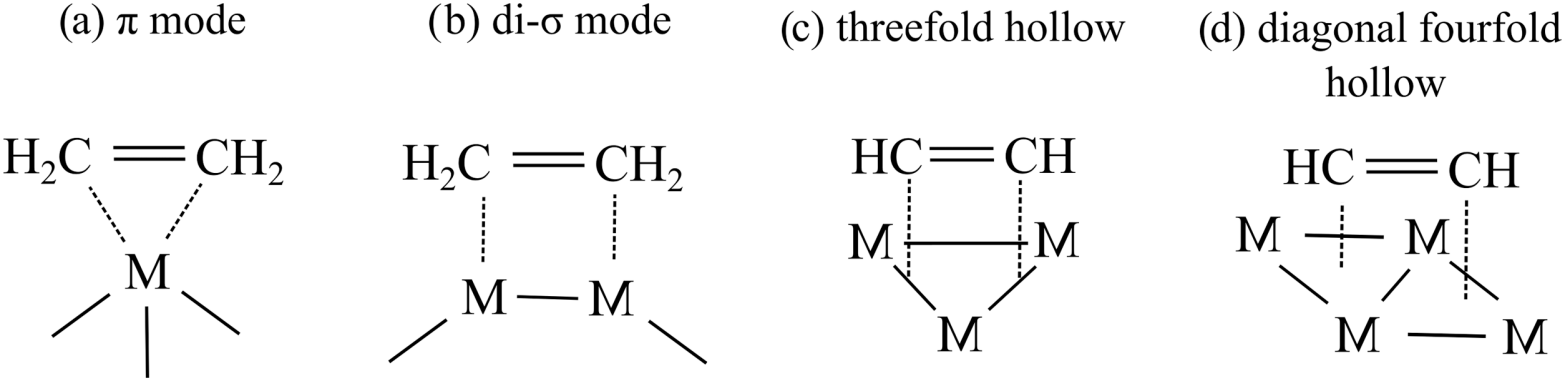
Binding Modes of Ethylene and Acetylene on Transition Metal Surfaces or Nanoparticles M represents a transition metal atom.

The adsorption of small, unsaturated hydrocarbons such as ethylene and acetylene on transition metal surfaces is also of great interest due to their importance in catalytic reactions. Their adsorption has been investigated on several different transition metal surfaces and clusters composed of Ni, Pd, Pt or Cu.^56−60^ Ethylene prefers two different binding modes, namely π- or di-σ-orientation, where the two carbon atoms bind to the same or to adjacent metal atoms, respectively (Scheme 2a,b). On copper clusters, ethylene adsorption was found to be selective for π-coordination.^61^ In the case of acetylene, it was observed that it adopts low-symmetry (*C*_1_) geometry on the Cu(110) surface,^59^ while threefold hollow and diagonal fourfold hollow adsorption modes have been suggested to be the most favorable on the Cu(100) surface (Scheme 2c,d).^62^ In contrast, it was found that π- or di-σ bindings of acetylene are more stable than the adsorption at the hollow sites on Cu*_n_* clusters with *n* = 10–15.^61^ In addition, the computations indicate that the adsorption strength (for both ethylene and acetylene) varies depending on the parity of the atoms, resulting in stronger binding to Cu*_n_* with *n* = 11,13,15 compared to the even-numbered neighbors.

However, despite their importance in several catalytic processes, our knowledge on the adsorption of ethylene and acetylene on iron surfaces, nanoparticles, and clusters remains incomplete. It has been observed experimentally that both ethylene and acetylene adsorb molecularly on the iron surface at 100 K, but thermally dissociate above room temperature.^63^ Small Fe cluster models representing different surface orientations of Fe bcc have been previously used to investigate different adsorption sites of acetylene.^64^ In the case of ethylene, computational studies involving small iron clusters have shown that π-orientation is favorable for Fe_2_ and Fe_3_ while di-σ-orientation provides stronger adsorption for Fe_4_.^65^ However, other computations do not indicate this relationship between the cluster size and adsorption configuration.^66^ To the best of our knowledge, hitherto, no computational or experimental data have been reported for acetylene and ethylene adsorption on larger iron clusters or nanoparticles.

In the synthesis of CNT by FCCVD, sulfur was found to be a crucial component, used as a promoter to increase the growth efficiency.^10−17,67^ In small quantities, sulfur helps to avoid the complete carbonization of the metallic surface, which could cause deactivation. Without sulfur, there is a significantly higher probability of iron nanoparticle catalyst deactivation due to carbon encapsulation (as illustrated in Scheme 1c,d).^28^ Furthermore, its promoter effect has also been observed in substrate-supported CCVD.^15,68,69^ Most commonly, sulfur is introduced as a powder or in the form of a sulfur-containing compound, such as thiophene or carbon disulfide, which decomposes on the surface of the growing iron nanoparticles, providing sulfur atoms on the catalyst surface.^70^ Its presence affects many stages of the CNT growth process.^67^ As sulfur resides on the iron nanoparticle surface, it reduces surface tension, which affects the growth of iron nanoparticles by collision. Moreover, it reduces the melting point of the nanoparticles, influencing carbon diffusion, which leads to CNT formation. By reducing the binding strength between the growing carbon cap and the iron nanoparticles, it helps carbon cap lift-off, which promotes CNT growth and inhibits catalyst deactivation.^16,71^ Above 1000 °C, sulfur begins to evaporate in the form of H_2_S, giving greater access to the catalyst surface for adsorption of carbon source, thereby facilitating the nucleation and growth of CNT.^14^ Thus, sulfur influences various structural properties of the synthesized CNTs, such as the diameter,^72^ wall numbers^12,73^ and crystallinity.^74−76^ Moreover, computations showed that the weakened binding of the growing CNT to the iron nanoparticle in the presence of sulfur enhances the growth rate.^71^ This leads to longer CNT products, which were observed experimentally.^15^

Additionally, sulfur plays an important role in other transition metal-catalyzed processes,^77^ including Fischer–Tropsch synthesis,^78,79^ ammonia synthesis,^80,81^ fluid catalytic cracking (FCC),^82^ and selective catalytic reduction (SCR).^83^ Even in small quantities, it greatly reduces the catalytic activity by adsorbing on the surface, thus poisoning the catalyst and affecting the conversion. Therefore, the adsorption of sulfur and the effect of sulfur coverage on catalytic activity have been widely investigated for several transition metal surfaces with different adsorbate molecules using DFT computations. It was found that sulfur only weakens CO adsorption in its immediate proximity on Pd(100) and Fe(100) surfaces.^84,85^ Because sulfur preferentially adsorbs on the hollow sites of the metal surface, it occupies multiple possible adsorption sites from the molecules.^85,86^ In the case of CH_*x*_ formation from synthesis gas, sulfur has been shown to weaken the adsorption of different species on Cu(111).^86^ However, increasing the sulfur content did not further destabilize the adsorption. It has also been shown that sulfur affects the kinetics of hydrogenation by increasing the activation barriers. Despite its poisoning effect on the catalytic performance, sulfur is often used as a promoter (even in combination with sodium or potassium^87,88^) in the iron-based Fischer–Tropsch process due to its tuning effect on the product selectivity by blocking chain growth.^89^

Although sulfur is a common additive in the FCCVD production of CNTs, its effect on the initial stages of the growth mechanism is still not fully understood. The goal of our work is to investigate the adsorption of two common precursors, acetylene and ethylene, on iron clusters and nanoparticles and the effect of sulfur. This models the nascent phase of the FCCVD process, where small nuclei of catalyst nanoparticles grow while the adsorption of sulfur and carbon sources can occur, as shown in Scheme 1b. In addition, the use of iron clusters or nanoparticles and the effect of sulfur are also of great interest in Fischer–Tropsch synthesis; thus, this study can offer valuable information about the impact of sulfur on the process.

## 2 Computational Details

### 2.1 Total Energy Computation and Geometry Optimization

All computations were carried out using the GPAW package in conjunction with the Atomic Simulation Environment.^90−92^ As dispersion effects can play an important role in adsorption, we used the C09-corrected version of the first-generation van der Waals density functional (C09-vdW-DF),^93−95^ which has been successfully used in our previous work to describe the interaction of carbon nanotube caps and catalyst nanoparticles.^71^ The Kohn–Sham equations were solved using the projector-augmented wave method (PAW) in which the Kohn–Sham orbitals were expanded in plane waves (PW) up to a cutoff energy of 500 eV.^96^ An electronic Fermi smearing of 0.1 eV was used, and the total energies were extrapolated to 0 K. All computations involved spin polarization. The self-consistent field energy convergence threshold was set to 10^–6^ eV per valence electron. A box with a size of 20 × 20 × 20 Å^3^ (25 × 25 × 25 Å^3^ for Fe_55_ systems) and only a single *k*-point (Γ-point) was considered.

The geometry optimization was performed using the FIRE algorithm^97^ until the magnitude of the force on every atom was less than 0.01 eV/Å (0.02 eV/Å for Fe_55_ and fcc(111) iron surface systems, which results in a negligible difference in total energy but saves significant computational time compared to the criteria of 0.01 eV/Å). A triple-zeta atomic basis set with polarization functions (tzp) was employed within the linear combination of atomic orbitals (LCAO) method, as it was found to provide an appropriate structural description while its computational demand is significantly reduced compared to that of the PW method.^98^ Further details of the computations are described in the Supporting Information (SI).

### 2.2 Adsorption Configurations

We systematically investigated acetylene and ethylene binding at different sites of Fe*_n_* (*n* = 3–10, 13, 55). First, the geometries of the clusters and adsorbates were separately optimized using the computational method described above. Then, an in-house developed program was applied to identify the different sites on the clusters and generate the initial structure of the Fe*_n_*–adsorbate adducts in the binding modes, as shown in Scheme 2. To obtain local minima, the geometries of the initial configurations were fully optimized. Thus, we have identified several possible structures for each Fe*_n_*–adsorbate adduct. Here, we will focus on the lowest energy configurations while all of the optimized structures can be found in Section 5 in the SI. We also performed molecular dynamics simulations to investigate the structural stability of the lowest energy isomers, as described in SI.

## 3 Results and Discussion

### 3.1 Properties of Iron Clusters

**Figure 1:**
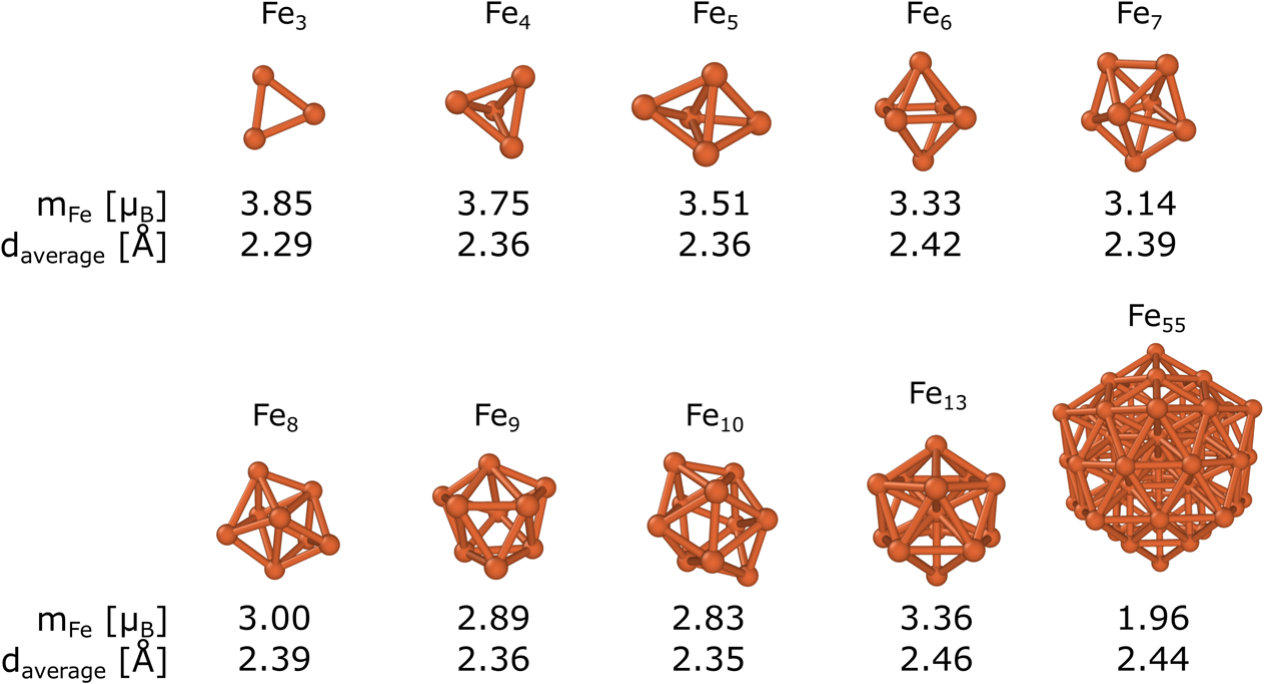
Optimized structures of Fe*_n_* (*n* = 3–10,13,55) clusters. *m*_Fe_ is the magnetic moment per Fe atom, and *d*_average_ is the average bond length in each system.

We considered clusters and nanoparticles of different sizes (Fe*_n_n* = 3–10, 13, 55) in our computations. The optimized structures are shown in Figure 1, along with their main structural and magnetic parameters. In the case of *n* = 3–10, we reproduced the cluster structures based on previous studies.^25,53^ For 13- and 55-atom particles, we used the icosahedral configuration as it was experimentally observed before.^26^ Also, an icosahedral 13-atom cluster was previously used to investigate the adsorption of different molecules on iron and other metals.^54,99^ The average bond length (*d*_average_ = 2.46 Å) and magnetic moment per Fe atom (*m*_Fe_ = 3.36 μ_B_) of Fe_13_ are in good agreement with the previously reported computational data (2.46 Å and 3.38 μ_B_, computed in ref (54)). For Fe_55_, *m*_Fe_ is noticeably reduced (*m*_Fe_ = 1.96 μ_B_) compared to the smaller clusters due to antiferromagnetic coupling between the surface iron atoms (*m*_Fe_ = 2.57 μ_B_) and the inner atoms (*m*_Fe_ = −0.35 μ_B_). Additionally, the inner shells consist of highly coordinated iron atoms, resulting in substantially smaller magnetic moments. The antiferromagnetic coupling has also been observed in a prior study though the magnetic moment per Fe atom is larger (*m*_Fe_ = 2.72 μ_B_).^100^

### 3.2 Acetylene and Ethylene Adsorption

We investigated the adsorption of acetylene and ethylene molecules by considering the numerous adsorption sites for each Fe*_n_* (*n* = 3–10, 13, 55). The binding energy was calculated as follows
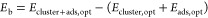
where *E*_cluster,opt_, *E*_ads,opt_, and *E*_cluster+ads,opt_ are the total energies of the gas-phase iron cluster or nanoparticle, adsorbate (acetylene or ethylene) and their adduct, respectively. Based on this definition, a negative binding energy indicates favorable binding.

**Figure 2:**
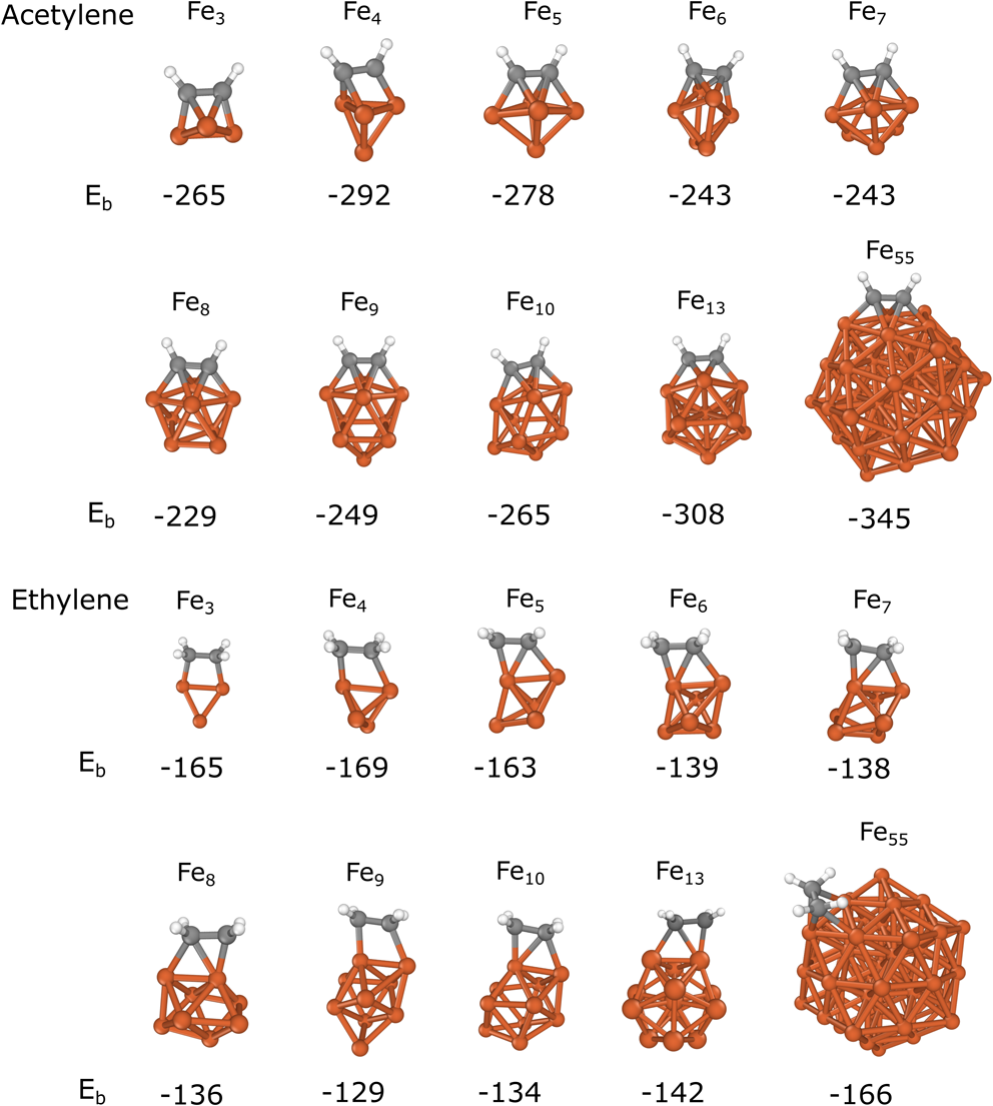
Most stable acetylene and ethylene adsorption sites on Fe*_n_* (*n* = 3–10, 13, 55). The calculated binding energies (*E*_b_) are given in kJ/mol.

The most stable structures and their corresponding binding energies are shown in Figure 2. For later reference of the structures, the formula of the iron cluster/nanoparticle with A for acetylene or E for ethylene is used (for instance, Fe_8_ + A means the lowest energy structure with acetylene adsorbed on Fe_8_, as shown in Figure 2). The calculated energies show significantly stronger binding for acetylene (in the range of −230 to −350 kJ/mol) than for ethylene (in the range of −130 to −170 kJ/mol) since acetylene interacts with triple bonds while ethylene coordinates with double C–C bonds on the surface of the iron clusters. Although there was no clear connection between the sizes of Fe*_n_* and the binding energies, the strongest adsorption was observed for Fe_55_. In both cases, the binding energies are −345 and −166 kJ/mol for Fe_55_ + A and Fe_55_ + E, respectively, similar to those on the fcc(111) iron surface (−375 kJ/mol for acetylene and −171 kJ/mol for ethylene; see Section 2 in the SI for further details). Furthermore, it is also obvious that the two adsorbate molecules favor different adsorption sites, which correlates with the difference between their unsaturated C–C bonds. Acetylene prefers to orient so the two carbon atoms are located above the hollow sites of the adjacent three-membered iron rings on the clusters or nanoparticles, which is denoted as a diagonal fourfold hollow adsorption mode (as illustrated in Scheme 2d). This is the binding mode with the highest coordination and a decreasing tendency is found in the binding strength as acetylene binds to the lower coordination sites on the iron clusters (di-σ or π mode, as summarized in Section 5 of the SI). Our molecular dynamics simulations show that the diagonal fourfold hollow configuration remains stable even at high temperatures (see Figure S5 in the SI). The Fe_3_ and Fe_4_ clusters are too small to accommodate this structure and thus the threefold hollow site is preferred (as shown in Scheme 2c). We found that the diagonal fourfold hollow configuration is also the most stable on the fcc(111) iron surface (see Figure S2 in the SI).

In contrast, ethylene preferentially binds in the di-σ mode on small clusters, where the adsorbate molecule is located above a Fe–Fe bond at the cluster edge (as depicted in Scheme 2b). However, in the case of Fe_13_ and Fe_55_, a new binding mode becomes favorable in which the carbon atoms are above the Fe_3_ ring while two hydrogens (one from each carbon atom) interact with the adjacent Fe atoms (tilted π configuration as shown for Fe_55_ + E in Figure 2). For Fe_13_, the binding energy of this site is slightly more positive (−131 kJ/mol) as in the most stable di-σ mode (−142 kJ/mol in Figure 2) while for Fe_55_, it becomes a more stable configuration (*E*_b_ = −166 and −151 kJ/mol for tilted π and di-σ orientations, respectively). This suggests that ethylene preferentially binds to lower coordination binding sites than acetylene due to the lower saturation of the C–C bond. However, the adsorbed ethylene can quickly dissociate with acetylene on the surface at high temperatures, leading to a change in the binding mode (Figure S5 in the SI). The icosahedron is encompassed by fcc(111) planes, and interestingly, the same tilted π binding mode was found to be the most favored on the bulk fcc(111) surface (see Figure S2 in the SI).

Both acetylene and ethylene are considered as precursors with low decomposition temperatures in CNT synthesis which can easily lead to catalyst deactivation if the injection temperature and length are not carefully set.^101^ In contrast, using methane, a precursor with a high decomposition temperature, offers a smaller chance of catalyst deactivation and greater flexibility in the process for successful CNT growth. Comparing the calculated binding energies of Fe_13_ + A (−308 kJ/mol) and Fe_13_ + E (−142 kJ/mol) with previous computations for methane adsorption on icosahedral Fe_13_ (−0.28 eV = −27 kJ/mol in ref (54)), methane binds significantly more weakly, while its decomposition temperature is higher (750 °C for methane, 440 °C for ethylene and 400 °C for acetylene). This suggests that a longer contact time between the growing catalyst nanoparticles and acetylene or ethylene precursor provides a faster growth rate for carbon cap formation, thus increasing the chance of catalyst deactivation than in the case of methane. This can explain the greater flexibility of the contact time and, thus, the injection length for successful CNT growth using methane compared with ethylene or acetylene.

Moreover, our results suggest that deactivation and growth rates can differ when acetylene or ethylene is used as the carbon source. The stronger binding of acetylene to the growing iron nanoparticles than that of ethylene, coupled with its lower decomposition temperature, provides a greater carbon supply for CNT growth. This results in a greater growth rate and, thus, longer CNT products with acetylene than ethylene. This correlation has been previously observed in the synthesis of CNTs using a mixture of acetylene and ethylene in different ratios as carbon sources.^102^

### 3.3 Effect of Sulfur on the Adsorption of Acetylene and Ethylene

**Figure 3:**
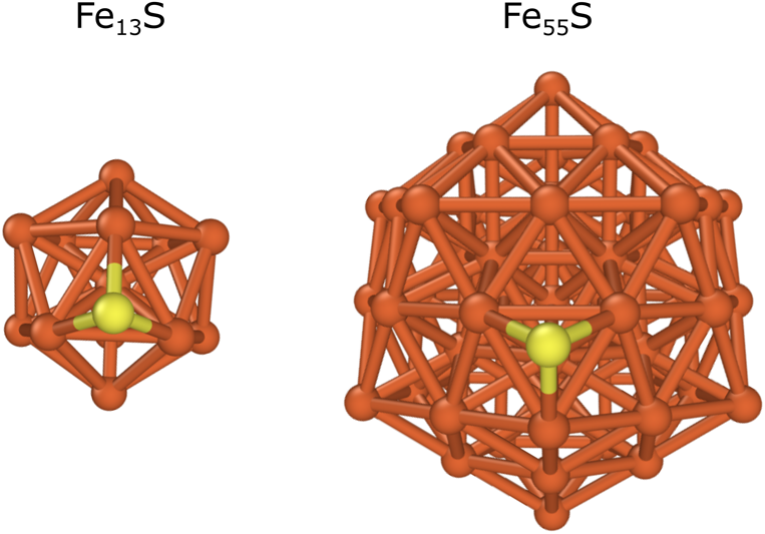
Optimized structures of Fe_13_S and Fe_55_S.

Fe_13_ and Fe_55_ nanoparticles were used to investigate the effect of sulfur, as these structures are large enough to host several different surface sulfur and adsorbate configurations. First, we studied the adsorption with a single sulfur atom placed on the cluster surface (Fe_13_S and Fe_55_S). Previous studies have shown that sulfur is the most stable in the hollow position on metal surfaces,^85,86^ and according to our computations, sulfur atoms are located above a three-membered ring on Fe_13_ or Fe_55_. The optimized structures are shown in Figure 3.

**Figure 4:**
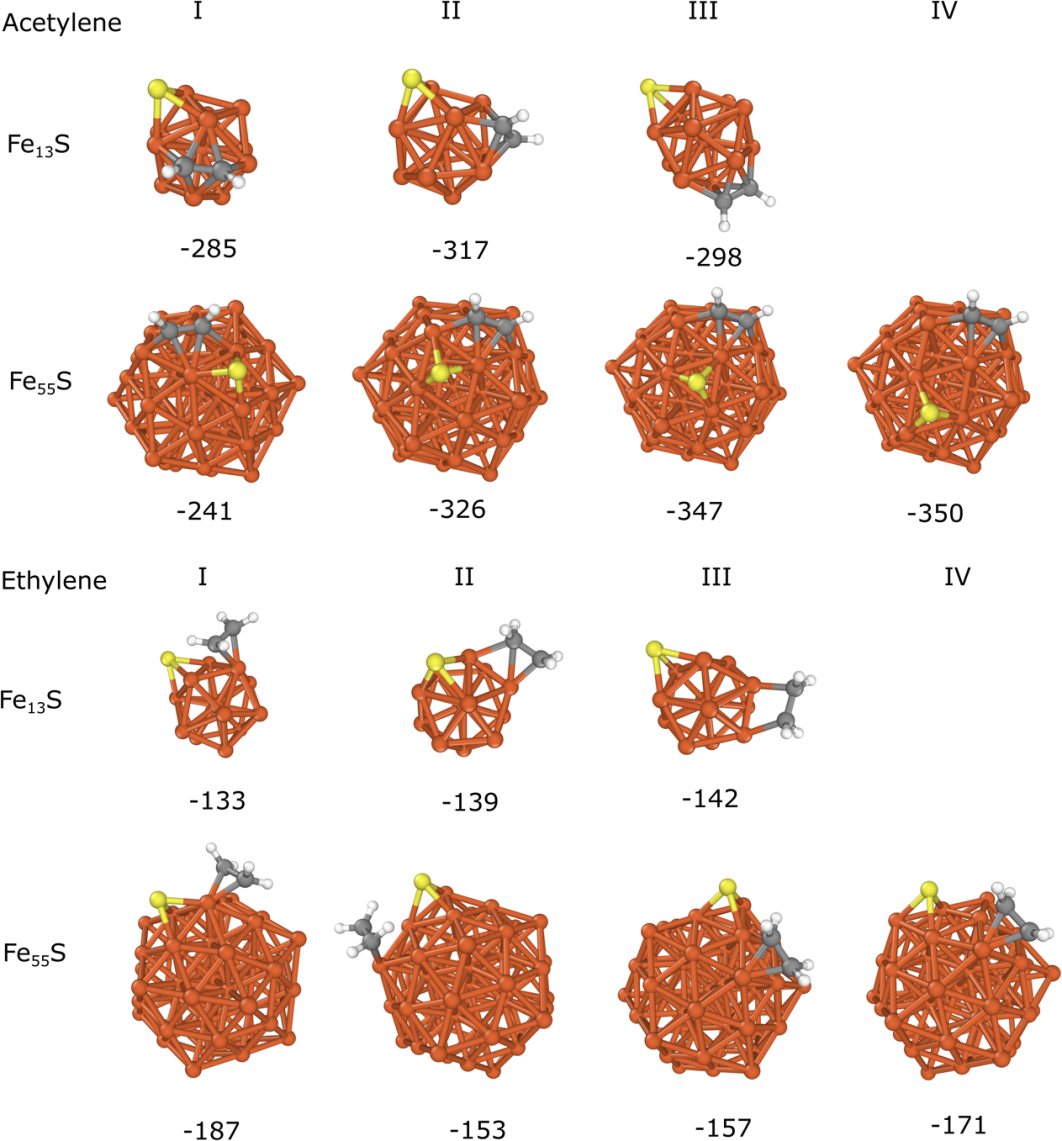
Adsorption of acetylene and ethylene on Fe_13_S and Fe_55_S with different sulfur–adsorbate molecule configurations. The calculated binding energies (*E*_b_) are shown in kJ/mol.

For both acetylene and ethylene adsorbates, the most stable adsorption site was selected, and several structures with different sulfur–adsorbate distances and configurations were considered. The optimized structures and the calculated binding energies are summarized in Figure 4. The previously used nomenclature was extended with Roman numerals to refer to the different structures in the case of the same cluster/nanoparticle and adsorbate. For instance, Fe_13_S + E(I) refers to the first structure of ethylene adsorbed on Fe_13_S with a binding energy of −133 kJ/mol. Based on these results, the effect of sulfur on adsorbate binding is significant only in the immediate vicinity of the adsorbate molecules. On Fe_13_S, the largest increase in the binding energy was observed for Fe_13_S + A(I) (*E*_b_= −285 kJ/mol) compared to Fe_13_ + A (*E*_b_= −308 kJ/mol). By increasing the distance between the acetylene and sulfur atom, the difference in the binding energy becomes only 10 kJ/mol (−317 and −298 kJ/mol for Fe_13_S + A(II) and Fe_13_S + A(III), respectively). In the case of ethylene, the binding energies depend only weakly on the sulfur position, and the differences in *E*_b_ are within the range of 10 kJ/mol (−133, −139 and −142 kJ/mol for Fe_13_S + E(I), Fe_13_S + E(II) and Fe_13_S + E(III), respectively) compared to Fe_13_ + E (*E*_b_= −142 kJ/mol). On Fe_55_S, acetylene shows an outstanding increase in binding energy in the Fe_55_S + A(I) configuration (*E*_b_= −241 kJ/mol), while the change is less significant for Fe_55_S + A(II) (*E*_b_= −326 kJ/mol) than sulfur-free Fe_55_ (*E*_b_= −345 kJ/mol). Although the relative coordination between acetylene and the sulfur atom is the same in Fe_55_S + A(I) and Fe_55_S + A(II), the difference in their structures is that in the case of Fe_55_S + A(I), acetylene and the sulfur atom are located on the same surface plane of the icosahedral structure, but they are on neighboring planes in Fe_55_S + A(II). This suggests that the effect of sulfur on the binding strength has a considerable steric repulsion contribution while electronic effects can play a minor role. For ethylene, the binding energies exhibit a smaller degree of change due to the presence of sulfur. In Fe_55_S + E(II) and Fe_55_S + E(III), steric repulsion between hydrogen and sulfur leads to an increase in the binding energy (−153 and −157 kJ/mol, respectively). In the case of Fe_55_S + E(I), our computation converged to a different magnetic state which can explain the stronger adsorption (−187 kJ/mol) compared to that of Fe_55_ + E (−166 kJ/mol). This is the only structure in which the magnitude of the binding energy suggests stronger binding of ethylene in the presence of sulfur. However, the typical temperatures used in CNT growth by the FCCVD technique are higher than the Curie temperature of iron; thus, magnetism is expected to play only a minor role in operando conditions. In the case of Fe_13_S + A(II), Fe_55_S + A(IV), and Fe_55_S + E(IV), the *E*_b_ values (−317, −350, and −171 kJ/mol, respectively) also indicate slightly stronger binding compared to the corresponding cases without sulfur, but the differences are less than 10 kJ/mol which could originate from the numerical inaccuracy of the computational method.

**Figure 5:**
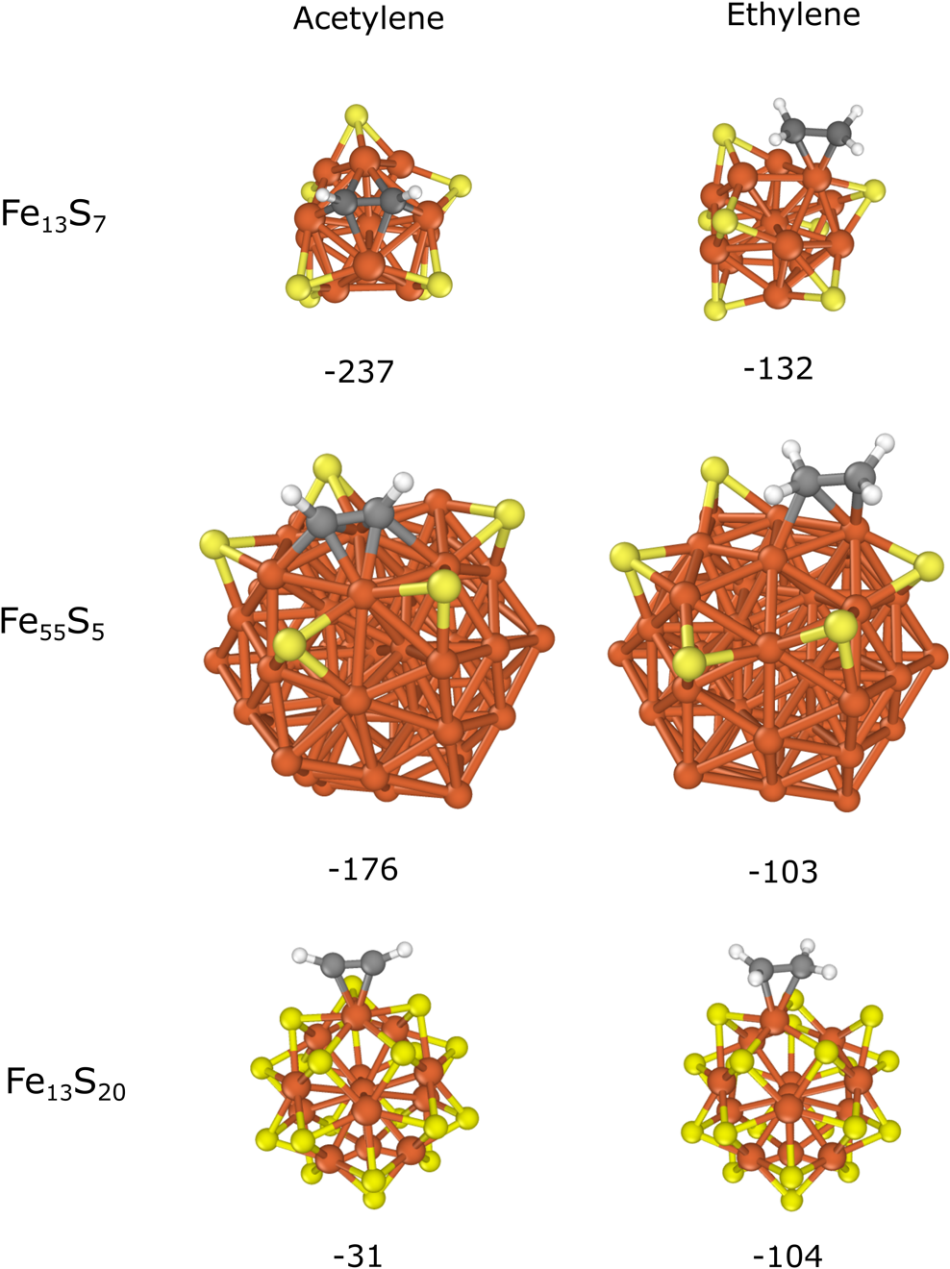
Acetylene and ethylene adsorption on Fe_13_S_7_, Fe_55_S_5_ and Fe_13_S_20_. The calculated binding energies (*E*_b_) are shown in kJ/mol.

Adsorption was also investigated with a higher surface sulfur content. The most stable binding modes of acetylene and ethylene were selected as in the case of Fe_13_ and Fe_55_ (shown in Figure 2) and different amounts of sulfur atoms were placed on the iron particles. Fe_13_S_7_ and Fe_13_S_20_ were chosen to represent partial and full surface coverage of the icosahedral Fe_13_, respectively, while Fe_55_S_5_ with only partial coverage around the adsorbate was applied, as farther sulfur atoms had a small effect on the binding (as shown in the case of Fe_55_S + A(IV) and Fe_55_S + E(IV) in Figure 4). Our choice of sulfur distribution for Fe_13_S_7_ and Fe_55_S_5_ is further explained in Section 3 of the SI. The optimized structures and the calculated adsorption energies are shown in Figure 5. The binding of acetylene to Fe_13_S_7_ (−237 kJ/mol) and Fe_55_S_5_ (−176 kJ/mol) is significantly weakened, though its configuration remains identical to those of Fe_13_ + A and Fe_55_ + A. In the case of ethylene, the di-σ mode on Fe_13_ changes to the π configuration on Fe_13_S_7_ due to the adjacent sulfur atoms. This also suggests a considerable steric effect of sulfur. Interestingly, the binding strength remains nearly the same (−132 kJ/mol) as that of Fe_13_S + E(I) when comparing their binding energies. In contrast, ethylene binds considerably weaker to Fe_55_S_5_ (−103 kJ/mol) than to Fe_55_ or Fe_55_S while its coordination also shifts due to the neighboring sulfur atoms.

Complete sulfur coverage of the surface (Fe_13_S_20_) inhibits the preferred binding sites of acetylene or ethylene, allowing only π coordination. This results in an even greater reduction in the binding strength of the adsorbates. Interestingly, in this case, the binding of acetylene becomes significantly weaker (−31 kJ/mol) than that of ethylene (−104 kJ/mol). This is because the π mode is considerably less favored than the di-σ coordination of acetylene on Fe_13_ while there is no strong preference in the case of ethylene (see Section 5 of the SI). Although the binding of acetylene and ethylene on Fe_13_S_20_ is still preferable based on their negative *E*_b_ values, they can easily detach from the surface due to the steric repulsion of the surrounding sulfur atoms, as observed in our molecular dynamics simulations (Figure S5). This suggests that complete sulfur coverage of the surface can even suppress the adsorption of acetylene or ethylene on the iron surface.

The above results show that sulfur exerts two effects on the precursor adsorption process. This blocks several adsorption sites from the precursor molecules, which decreases the catalytic activity of the particles. This has been previously classified as the geometric effect of sulfur poisoning.^103^ Combining the geometric effect with sulfur’s behavior of high dispersion on the surface (as shown in Figure S4), the elongation of carbon chains is hindered which results in higher selectivity for shorter hydrocarbons (C_2_–C_4_ olefins) in the Fischer–Tropsch process.^87,88^ Furthermore, it can also influence CNT nucleation in the FCCVD process. In the early stages of iron nanoparticle formation, the precursor adsorption and, thus, the carbon cap growth on the nanoparticle surface could be hindered in the presence of sulfur, which inhibits the encapsulation of catalyst nanoparticles by a carbon shell in the lower-temperature zone of the reactor. Later, at higher temperatures, the sulfur evaporates, and the nanoparticles are active for CNT growth.^14^ Thus, by preventing the low-temperature catalyst nanoparticle deactivation, sulfur can promote CNT growth.^28^

In addition, sulfur also reduces the binding strength of the adsorbate in the neighboring sites, which has been referred to as the electronic effect.^103^ Our computations show that this effect is combined with a substantial steric repulsion between sulfur and the adsorbate, based on a comparison of the binding energies of Fe_55_S + A(I) and Fe_55_S + A(II). It has been found that sulfur tends to be distributed on iron particles^72^ (which was also found in our computations summarized in Figure S4), which can strongly decrease the binding strength of adsorbates on the whole surface. Moreover, our preliminary computations indicate that sulfur even inhibits the dissociation of precursor molecules (see Section 4 of the SI for further explanation). This can slow down the decomposition rate and the growth of the carbon cap, which can result in the growth of larger catalyst nanoparticles at the beginning of the FCCVD process.^72^ Thus, the reduced catalytic activity can increase the size of the catalyst nanoparticles, leading to CNTs with larger diameters and multiple walls. This effect has been observed in previously reported experiments.^10,12,14,73,104^

### 3.4 Charge and Energy Analysis

To investigate the electronic structure effects on acetylene and ethylene binding and to understand the effect of sulfur, atomic charges were computed using Bader’s atoms in molecules (AIM) method, and the charge density difference (CDD) was also determined. In the case of the Fe_5_, Fe_13_, and Fe_55_ particles, the most stable structures (as shown in Figure 2) were used. For analysis in the presence of sulfur, the closest sulfur–adsorbate configurations for the Fe_13_S (namely, Fe_13_S + A(I) and Fe_13_S + E(I) in Figure 4) and Fe_13_S_7_ structures (as shown in Figure 5) were selected. Moreover, the binding energies were further decomposed into interaction and deformation energies. The interaction energy (*E*_int_) was calculated as
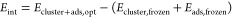
where *E*_cluster,frozen_ and *E*_ads,frozen_ are the total energies of the gas-phase iron cluster or nanoparticle and adsorbate (acetylene or ethylene) in the frozen geometry of their adduct, respectively. The deformation energy of the iron cluster/nanoparticle (*E*_def,Fe/FeS_) or adsorbate (*E*_def,ads_) refers to the energy difference between the frozen and relaxed structures. The sum of deformation and interaction energies yields the following binding energies
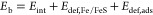


**Figure 6:**
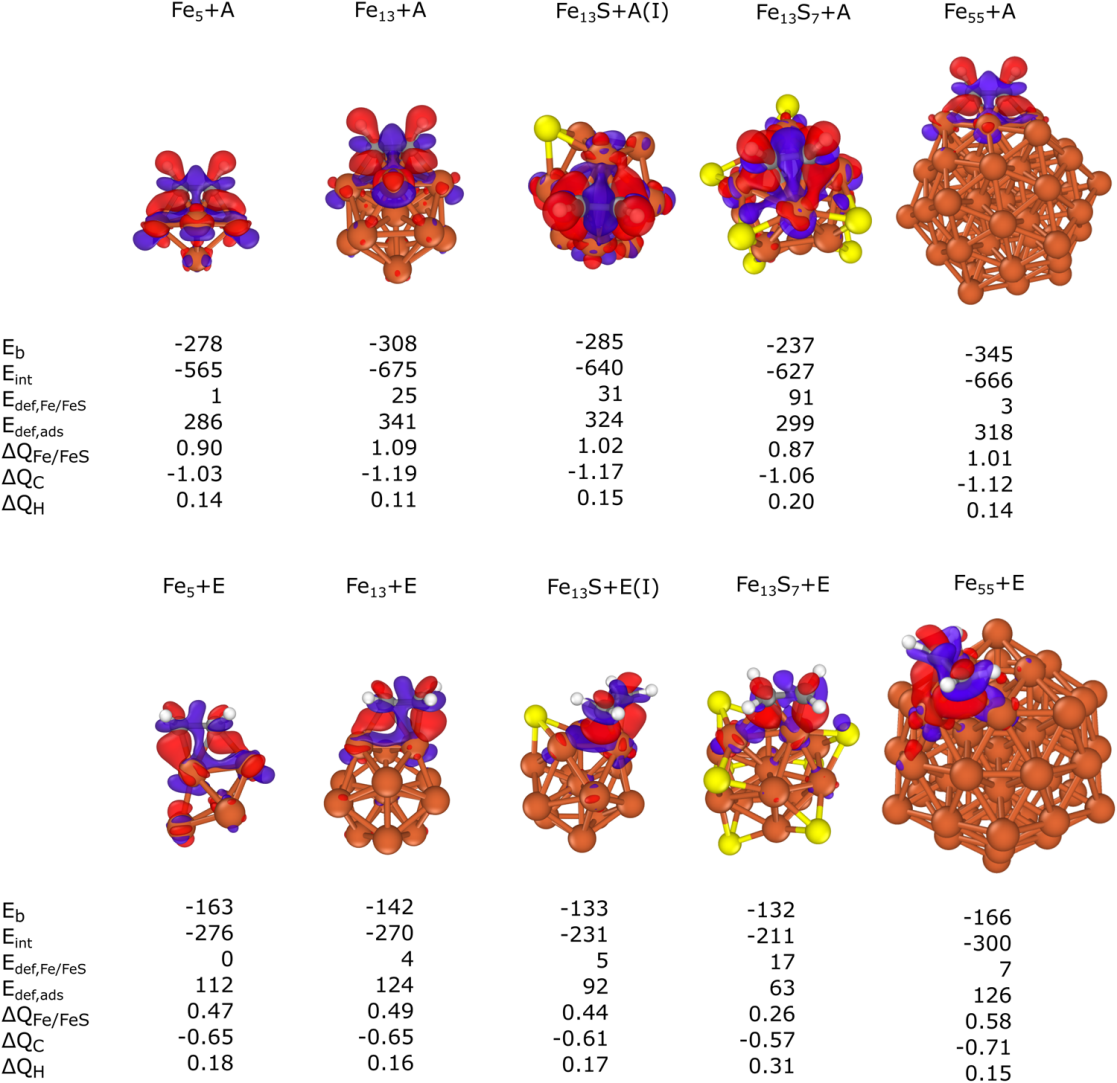
Energy decomposition, Bader, and charge density difference (CDD) analysis of acetylene and ethylene adsorption on Fe_5_, Fe_13_, Fe_13_S, Fe_13_S_7_, and Fe_55_. The calculated binding (*E*_b_), interaction (*E*_int_), and deformation (*E*_def_) energies are shown in kJ/mol, while Δ*Q*_Fe/FeS_, Δ*Q*_C_, and Δ*Q*_H_ are the total Bader charges for each atom type.

The results are shown in Figure 6. Δ*Q*_Fe/FeS_, Δ*Q*_C_, and Δ*Q*_H_ are the cumulated Bader charges (calculated by subtracting the Bader population from the atomic number for each atom) for either the atoms within the cluster/nanoparticle (Fe/FeS) or the carbon/hydrogen (C/H) atoms present in the adsorbate. A positive (negative) Δ*Q* indicates cationic (anionic) behavior. In all cases, Δ*Q*_Fe/FeS_ has a positive value, which indicates electron transfer to the adsorbate molecules from the cluster or nanoparticle. Based on the CDD figures, the adsorption may affect the partial population of the *d* atomic orbitals of the nearby iron atoms and the π* orbital of acetylene or ethylene. This suggests a donor–acceptor interaction between the occupied *d* atomic orbitals of the iron atoms and the unoccupied π* orbital of the adsorbate molecules. The greater absolute values of Δ*Q*_Fe/FeS_ and Δ*Q*_C_ for acetylene also confirm stronger binding to iron clusters than in the case of ethylene. It can also be observed that *E*_def,Fe/FeS_ and *E*_def,ads_ are greater for acetylene than for ethylene, highlighting the differences in their preferred binding modes. While ethylene undergoes only minor distortion from its planar structure to bind to iron clusters or nanoparticles, acetylene’s linear structure is significantly distorted, leading to greater deformation energy. Furthermore, the binding of acetylene tends to cause more significant structural distortions within the iron cluster or nanoparticle, primarily due to its higher coordination in the diagonal fourfold hollow mode, as opposed to the di-σ or tilted π binding modes of ethylene. The only exception is the case of Fe_55_, where *E*_def,Fe/FeS_ is slightly smaller for acetylene (3 kJ/mol) than for ethylene (7 kJ/mol), but both are negligibly small. Although there is no discernible relationship between *E*_b_ or *E*_int_ and the extent of electron transfer, there is a notable correlation between *E*_def,ads_ and electron transfer: the greater the deformation in the adsorbate, the more substantial the electron transfer. This can be explained by the larger extent of the C–C π bond weakening with greater *E*_def,ads_, which can then contribute to stronger binding and, thus, larger charge transfer between the adsorbate and the cluster.

Interestingly, Δ*Q*_Fe/FeS_ and the magnitudes of *E*_b_ and *E*_int_ decrease with increasing sulfur coverage for both adsorbates in the case of Fe_13_S_*x*_ clusters (namely, Fe_13_, Fe_13_S, and Fe_13_S_7_). For acetylene, Δ*Q*_Fe/FeS_ changes from 1.09 (Fe_13_ + A) to 0.87 (Fe_13_S_7_ + A) while it drops from 0.49 (Fe_13_ + E) to 0.26 (Fe_13_S_7_ + E) in the case of ethylene. This suggests a reduced electron transfer from the clusters to the adsorbates with higher sulfur concentration. However, the reduction might be partly compensated by electron transfer from the hydrogen to the carbon atoms as Δ*Q*_H_ is greater with increasing sulfur coverage for both adsorbates. It can also be noted that the deformation of the adsorbates becomes less significant with greater sulfur coverage as *E*_def,ads_ decreases from 341 kJ/mol (on Fe_13_) to 299 kJ/mol (on Fe_13_S_7_) for acetylene, while a reduction from 124 to 63 kJ/mol is observed on the same clusters in the case of ethylene.

As both *E*_b_ and *E*_int_ of acetylene or ethylene show the same tendency with increasing sulfur surface content, there is a direct correlation between the binding strengths of the adsorbates and the sulfur coverage of the surface of the catalyst particles.

## 4 Conclusions

Here, we presented a systematic density functional theory study on the adsorption of acetylene and ethylene on iron clusters and nanoparticles with Fe*_n_* (*n* = 3–10,13,55). We found that the binding of ethylene is significantly weaker than that of acetylene. Furthermore, ethylene prefers the di-σ adsorption mode for small iron clusters, but a different configuration becomes more favorable on Fe_55_, which is a tilted π mode so that the hydrogen atoms can interact with the adjacent iron atoms. In the case of acetylene, the preferred configuration is the diagonal fourfold hollow mode. To further investigate the interaction, Bader’s atoms in molecules (AIM) analysis and charge density difference (CDD) were used, which showed electron transfer from iron clusters or nanoparticles to the adsorbate molecule in both cases. The analysis suggests that electron transfer occurs mainly from the occupied d orbitals of the iron cluster to π* of the adsorbate. Furthermore, the effect of sulfur on adsorption was also investigated with different sulfur and adsorbate molecular configurations in the case of Fe_13_ and Fe_55_. The results showed that sulfur weakens the strength of adsorption only in the immediate proximity of the adsorbate, and the effect is mainly steric, while electronic effects playing only a minor role. However, the dense surface coverage of sulfur can significantly reduce both the number of adsorption sites and the strength of adsorption, which strongly affects the catalytic activity of the iron clusters or nanoparticles. This can promote catalyst nanoparticle growth and inhibit their carbon encapsulation, which would lead to deactivation at the early stage of carbon nanotube (CNT) nucleation in the floating catalyst chemical vapor deposition method. As sulfur evaporates from the catalyst nanoparticle surface at higher temperatures, its influence diminishes in the later phases of CNT growth. Additionally, this can account for the experimental observation of increased selectivity for shorter (C_2_–C_4_) olefins in Fischer–Tropsch synthesis. Thus, we believe that these computational results help understand the catalyst poisoning effect of sulfur, which also has a beneficial impact on several catalytic processes.

## Supporting Information Available

Method verification, the adsorption on Fe fcc(111) surface, sulfur distribution on Fe_13_, molecular dynamics simulation of adsorption on Fe_13_S_*x*_ (*x* = 0,1,7,20), all optimized adsorption modes on Fe_*x*_ (*x* = 3–10,13,55), and atomic positions of the most stable modes (PDF)

## Abbreviations

AIM: atoms in molecule
C09-vdW-DF: C09-corrected version of the first-generation van der Waals density functional
CNT: carbon nanotube
CDD: charge density difference
COVP: complementary occupied-virtual pairs of localized orbitals
DFT: density functional theory
FCC: fluid catalytic cracking
FTS: Fischer–Tropsch synthesis
PAW: projector-augmented wave method
PW: plane wave
