# Supplementary material for: Acetylene and Ethylene Adsorption during Floating Fe Catalyst Formation at the Onset of Carbon Nanotube Growth and the Effect of Sulfur Poisoning: a DFT Study
Source: Inorg Chem. 2024 Jul 10;63(29):13624–35. doi: 10.1021/acs.inorgchem.4c01830 (PMC11270998; doi:10.1021/acs.inorgchem.4c01830)
Supplement: Supplementary file 1 — ic4c01830_si_001.pdf [file ic4c01830_si_001.pdf]

Supporting information
for
Acetylene and ethylene adsorption during floating Fe catalyst formation at the onset of carbon nanotube growth and the effect of sulfur poisoning: a DFT study

Balázs Orbán[1], Tibor Höltzl[1,2,3]

1. Budapest University of Technology and Economics. Szent Gellért tér 4. H1111. Budapest. Hungary
2. MTA-BME Computation Driven Research Group. Szent Gellért tér 4. H1111. Budapest. Hungary
3. Furukawa Electric Institute of Technology. Késmárk utca 28/A. H1158. Budapest. Hungary

* Corresponding author.
E-mail address: tibor.holtzl@furukawaelectric.com
Postal address: Késmárk utca 28/A, H-1158, Budapest, Hungary

Table of contents

1. Method verification 2
2. Adsorption on Fe fcc(111) surface 4
3. Sulfur distribution on Fe_{13} 5
4. Molecular dynamics simulation of acetylene and ethylene on $Fe_{13}S_X$ 6
5. All optimized adsorption modes on each iron cluster 10
6. Atomic positions of the optimized structures 23
References 52

1. Method verification

The computational method was verified in the case of two different binding modes of acetylene on Fe_{13} (**Figure S1**). For the verification of DFT functional, structures were optimized using plane-wave method using cutoff energy of 500 eV. Four different DFT functionals were considered: PBE, PBE with D3 empirical dispersion correction (PBE-D3), first generation van der Waals density functional (vdW-DF) and its C09 corrected version (C09-vdW-DF). Other details of computations were the same as explained in Section 2 of the manuscript. The calculated binding energies are summarised in **Table S1**. For all the four functionals, the binding energies show stronger interaction in the case of II comparing to structure I. Although the difference in the binding energy of I and II slightly differs for the different functionals, they describe the show similar tendencies for the binding modes. Thus, we used C09-vdW-DF in our computations as it has been shown to describe dispersion effects well for interaction with transition metal surfaces in our previous work. [1]

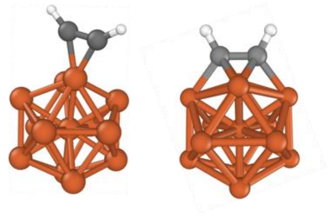

I II

Figure S1. Considered binding modes of acetylene on Fe_{13} for method verification.

Table S1. Calculated binding energies (E_b) in kJ/mol using different DFT functionals.

E_b [eV]	PBE	PBE-D3	vdW-DF	C09-vdW-DF
**I**	-147	-159	-123	-180
**II**	-249	-251	-205	-309
**Difference**	-102	-92	-82	-129

Expanding the wavefunctions in plane-waves can be computationally expensive, especially for non-periodic systems with vacuum regions. Thus, we investigated the possibility to apply linear combination of atomic orbitals (LCAO) to expand the wavefunctions in geometry optimization using triple-zeta basis set with polarization functions (TZP). The structures of I and II were optimized with both PW and LCAO methods and the binding energies were then calculated using the final energies in the case of PW optimization (PW opt), LCAO optimization (LCAO opt). Also, a PW energy computation was performed on the structures optimized by LCAO method (LCAO opt/PW sp). Results are summarized in **Table S2**. The calculated binding energies in the case of PW opt and LCAO opt differ significantly which indicate the inaccuracy of the LCAO method in energy computation due to the basis set superposition error. However, the results of LCAO opt/PW sp show negligible differences

comparing to those using PW optimization, which suggest that LCAO method is adequate for geometry optimization. Therefore, we applied LCAO method in geometry optimization while energy computation was performed with PW method to calculate accurate binding energies.

Table S2. Calculated binding energies (E_b) in kJ/mol using different optimization strategies with C09-vdW-DF functional. Cutoff energy for plane-wave (PW) expansion is 500 eV while triple-zeta basis set with polarisation functions were used in LCAO method.

**E_b [eV]**	**PW opt**	**LCAO opt/PW sp**	**LCAO opt**
**I**	-180	-178	-233
**II**	-309	-308	-385

2. Adsorption on Fe fcc(111) surface

The structures and binding energies of acetylene and ethylene to fcc(111) iron surface were computed using an orthogonal 4x4x3 slab model. The computations were periodic in two directions (along the surface) while a vacuum of 10 Å was applied in the non-periodic direction to avoid the interaction between the replicas of the system. The most stable structures with their binding energies are shown in **Figure S2**. For both adsorbates, the binding to the surface is stronger than to any of the clusters or nanoparticle. The binding mode of acetylene on the surface is the same as on the clusters (diagonal fourfold hollow) while ethylene has slightly different structure to that on Fe_{55} (tilted π configuration) as the two hydrogens closer to the surface are from the same carbon atom. The similar binding energies on Fe_{55} and fcc(111) can also be explained by the fact that the orientation of surface Fe atoms is the same. $d_{avarage}$ of Fe-Fe bonds is almost same (2.44 and 2.43 for Fe_{55} and fcc(111), respectively) which suggests identical adsorption sites on the surface of the two Fe structure.

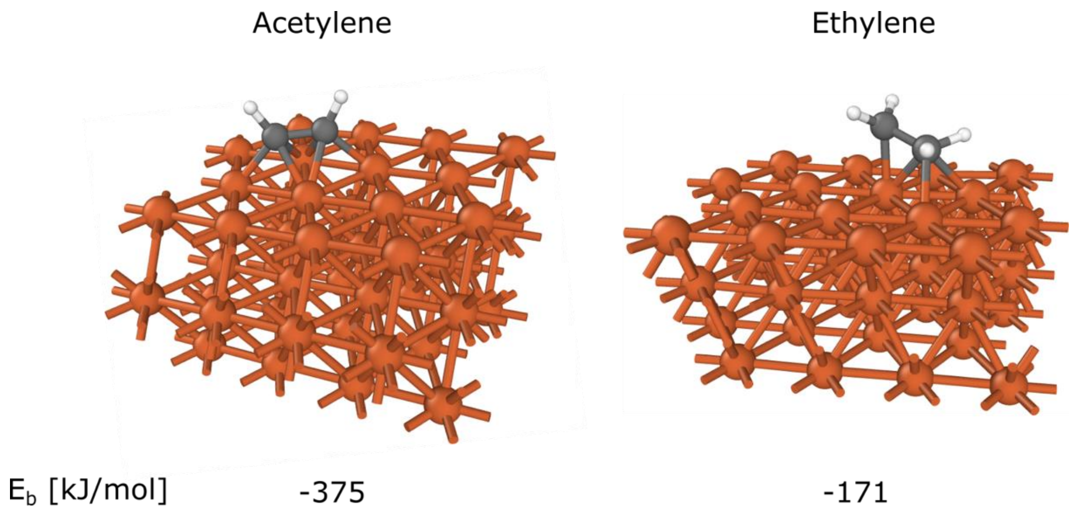

Figure S2. Optimized structure of acetylene and ethylene on Fe fcc(111) surface and their binding energies in kJ/mol.

3. Sulfur distribution on Fe_{13}

To select a sulfur distribution on $Fe_{13}S_7$ and $Fe_{55}S_5$ that realistically models the sulfur coverage, we systematically investigated the relative configurations of two sulfur atoms on Fe_{13} cluster. The optimized structures along with their relative energies are summarized in **Figure S3**. The results suggest that sulfur prefers to disperse on the surface while highest energy belongs to the configuration in which sulfur atoms are on adjacent planes of the icosahedral cluster (structure I). Although the lowest energy configuration is structure II, farther sulfur-sulfur distance as in the case of structure III and V does not increase their energy significantly.

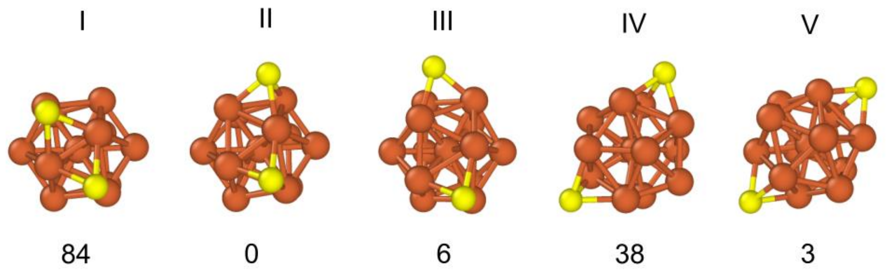

Figure S3. Optimized structures and relative energies in kJ/mol of $Fe_{13}S_2$ clusters with different sulfur configurations.

We further investigated sulfur distribution in the case of $Fe_{13}S_7$. Three structures were computed as shown in **Figure S4**. In structure *I* all sulfur atoms are located on neighboring planes of the icosahedral cluster while sulfur atoms are more and more distributed in the case of structure *II* and *III*. In structure *IV* none of the sulfur atoms is on adjacent planes and many of them have the same relative configuration as in structure *II* of $Fe_{13}S_2$. Their relative energies indicate that the more dispersed sulfur atoms are on the cluster surface, the lower the energy is. Thus, we selected structure *IV* to study the binding of acetylene and ethylene on $Fe_{13}S_7$. In the case of $Fe_{55}S_5$, a similar approach was used but only partial coverage was considered in which sulfur atoms were not placed above adjacent three membered rings of the icosahedral planes and follow the same relative configuration as in structure *II* of $Fe_{13}S_2$.

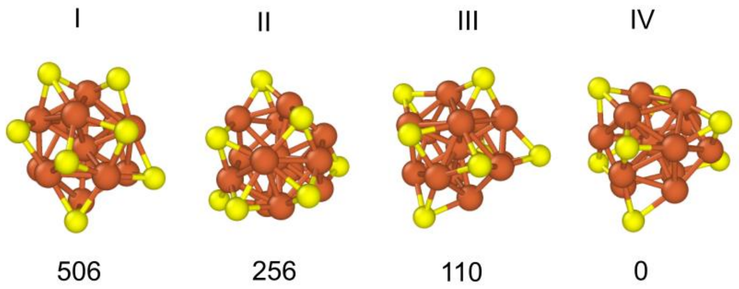

Figure S4. Optimized structures and relative energies in kJ/mol of $Fe_{13}S_7$ clusters with different sulfur configurations.

4. Molecular dynamics simulation of acetylene and ethylene on $Fe_{13}S_X$

Ab initio molecular dynamics (MD) simulations were also performed to study the behavior of acetylene and ethylene binding to iron clusters with and without sulfur at high temperature. Fe_{13}+A and Fe_{13}+E structures were chosen for sulfurless simulations while $Fe_{13}S$+A (I), $Fe_{13}S$+E (I), $Fe_{13}S_7$+A, $Fe_{13}S_7$+E, $Fe_{13}S_{20}$+A and $Fe_{13}S_{20}$+E were selected as sulfur containing systems. Total energy was computed with the same criteria as in geometry optimization. The Langevin thermostat was applied with simulation temperature of 1000 °C. Timestep was set to 0.5 fs. Snapshots of the simulations are summarized in **Figure S5**.

Based on the MD results acetylene on Fe_{13} remains in the same binding mode (diagonal fourfold hollow) after 4 ps. Although it slightly moves along the surface (switching from one hollow site to another), no considerable structural change was observed. On the other hand, ethylene starts to decompose after 0.5 ps by dissociating one of its hydrogens. Losing both hydrogen atoms after 1 ps, the acetylene also changes its binding from di-σ to diagonal fourfold hollow mode. Besides the behavior of the adsorbates, Fe_{13} cluster stays in its icosahedral structure which indicates its stability even at high temperature.

Acetylene behaves similarly on $Fe_{13}S$ as in the case of Fe_{13}. However, due to the proximity of the adsorbate and the sulfur atom, the sulfur atom migrates somewhat farther on the surface from the acetylene and change the configuration to the one of $Fe_{13}S$+A (II). This way the effect of sulfur on weakening the binding becomes negligible based on the calculated binding energies of Fe_{13}+A (E_b= -308 kJ/mol), $Fe_{13}S$+A (I) (E_b= -285 kJ/mol) and $Fe_{13}S$+A (II) (E_b= -317 kJ/mol). In the case of ethylene binding to $Fe_{13}S$, the dissociation does not begin after 4 ps and the structure remains very similar to the initial. Sulfur – adsorbate distance barely changes which suggests that there is negligible steric repulsion between them. This is in good agreement with the calculated binding energies of Fe_{13}+E (E_b= -142 kJ/mol) and the different $Fe_{13}S$+E binding configurations (E_b is -133, -139 and -142 kJ/mol for I, II and III, respectively).

As the sulfur coverage is further increased ($Fe_{13}S_7$), the adsorbates move less freely on the surface. Due to the significant steric and electronic repulsion, some neighboring sulfur atoms move a somewhat farther from the adsorbates while the displacement of acetylene and ethylene on the cluster surface is minimal compared to their initial position. This suggests that the active sites on the growing iron catalyst for precursor binding is limited due to the presence of sulfur. Although these MD results show acetylene and ethylene can still bind to the catalyst surface at this sulfur coverage, the calculated binding energies of $Fe_{13}S_7$+A (-237 kJ/mol) and $Fe_{13}S_7$+E (-132 kJ/mol) indicates the weakening of the active sites. However, the complete

sulfur coverage of the surface ($Fe_{13}S_{20}$) makes the binding of the adsorbates unstable which causes their detachment from the surface after 1 picosecond. This indicates that the complete sulfur coverage of the catalyst surface can even suppress the adsorption of acetylene and ethylene on the growing iron nanoparticle at the onset of CNT growth.

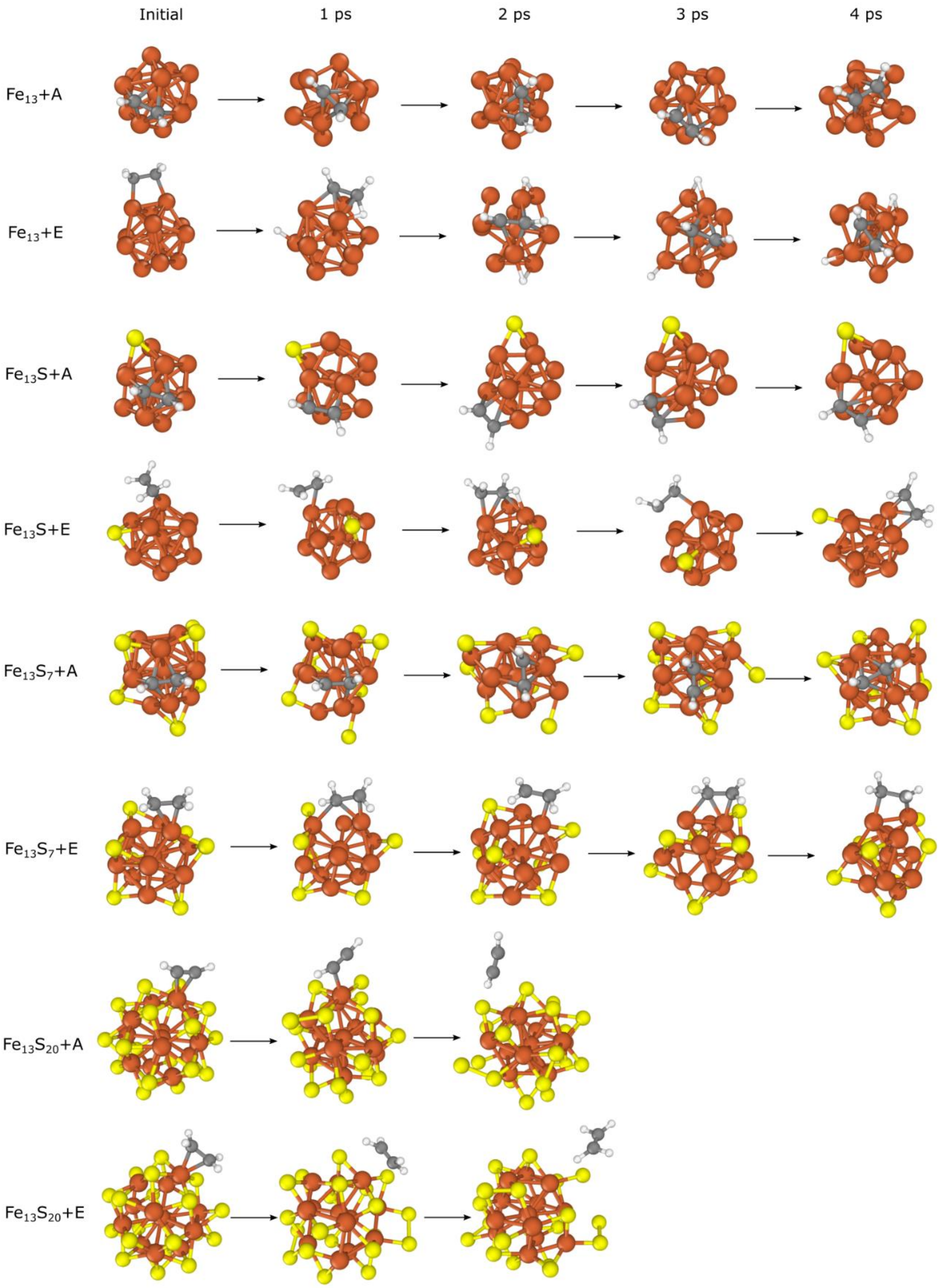

Figure S5. Snapshots of molecular dynamics simulation of acetylene and ethylene binding to $Fe_{13}S_x$ (x=0,1,7,20) at 1000 °C.

We also performed MD simulations on Fe_{13} and $Fe_{13}S_7$ clusters surrounded by 6 acetylene or ethylene molecules. The initial setup of these simulations is illustrated in **Figure S6 a)** in the case of Fe_{13} with acetylene and ethylene. Simulations were carried out for 4 picoseconds with the same computational method as described above. In **Figure S6 b)** snapshots of the clusters with the adsorbed molecules are shown at each picosecond.

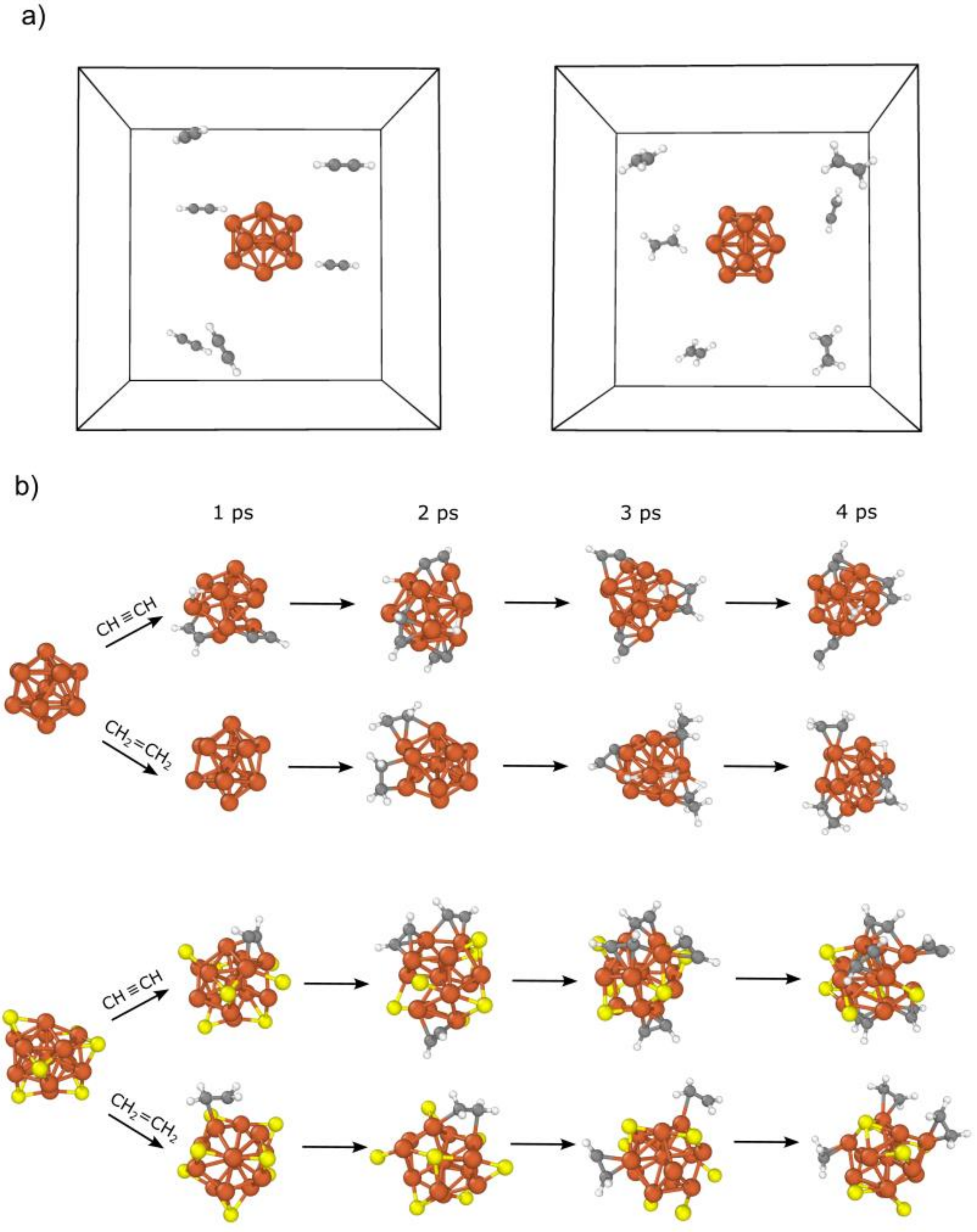

Figure S6.a) Initial setup of Fe_{13} surrounded by 6 acetylene (left) and ethylene (right) molecules. b) Snapshots of molecular dynamics simulations on acetylene and ethylene molecules binding to $Fe_{13}S_x$ (x=0,7) at 1000 °C.

Interestingly, ethylene or acetylene starts to decompose on bare Fe_{13}, but in the case of $Fe_{13}S_7$ the decomposition is hindered. As the molecular dynamics timescale is short, we further investigated the decomposition using static computations. The decomposition begins with the C-H bonds cleavage. Based on our MD trajectories, several structures were considered in which the dissociating hydrogen atom moves to an adjacent plane of the icosahedral structure. In **Figure S7** the optimized structures with the lowest energy are summarized along with their energy change comparing to the cluster-adsorbate adduct. For both acetylene and ethylene sulfur increases the energy change significantly, suggesting that the dissociation is less likely to occur with sulfur coverage. Moreover, a negative energy change is only observed in the case of Fe_{13}+E which is in good agreement with our MD simulations shown in **Figure S5** as the dissociation of the adsorbate only occurred in Fe_{13}+E system. These results, combined with the calculated binding energies, suggest that sulfur can hinder the decomposition of precursor molecules at the onset of FCCVD process. This slows down the carbon cap formation which can help to avoid carbon encapsulation of catalyst nanoparticles. In addition, it allows longer retention for catalyst nanoparticle growth, thus CNTs with larger diameter can form. Although decomposition of acetylene and ethylene is out of the scope of our study (adsorption of acetylene and ethylene), these MD results propose similar conclusions as drawn from our static computations on the adsorption of acetylene and ethylene.

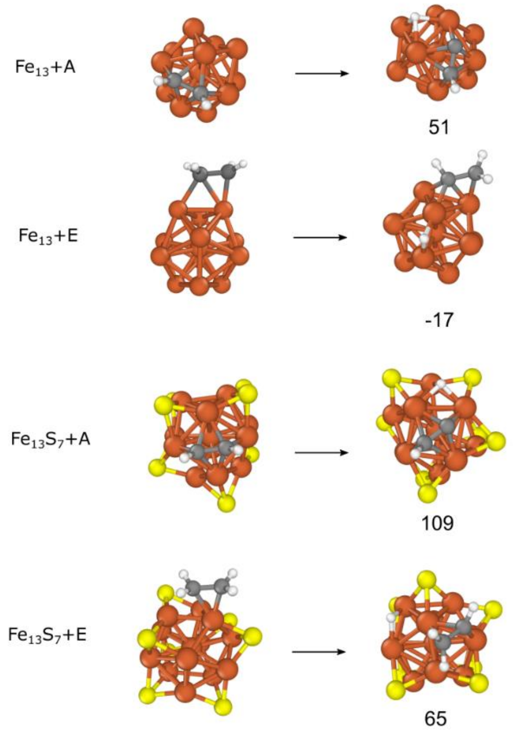

Figure S7. Optimized structures of acetylene and ethylene binded to Fe_{13} and $Fe_{13}S_7$ clusters and their dissociated structures by losing one hydrogen. The energy changes with the hydrogen dissociation are shown in kJ/mol.

5. All optimized adsorption modes on each iron cluster

For all structures, E_b (PW) and E_b (LCAO) were calculated following the computational method of LCAO opt/PW sp and LCAO opt, respectively, as described in Section 1 of the SI. The cutoff energy for PW expansion was set to 500 eV while triple-zeta basis set with polarization functions were used in LCAO method.

Fe_3

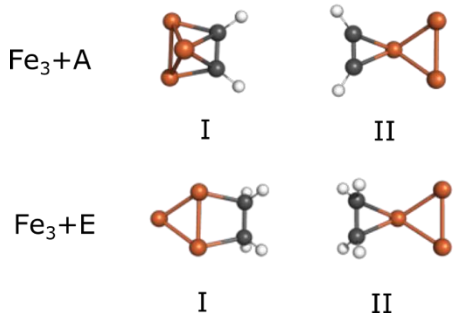

Figure S8. Optimized structures of acetylene and ethylene binded to Fe_3.

Table S3. Calculated binding energies (E_b) in kJ/mol of acetylene and ethylene binded to Fe_3.

Structure	E_b (PW)	E_b (LCAO)
Fe_3+A		
I	-265	-307
II	-194	-239
III	-220	-282
Fe_3+E		
I	-165	-218
II	-149	-197

Fe_4

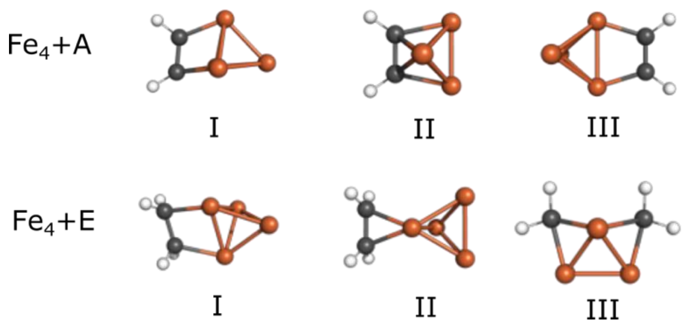

Figure S9. Optimized structures of acetylene and ethylene binded to Fe_4.

Table S4. Calculated binding energies (E_b) in kJ/mol of acetylene and ethylene binded to Fe_4.

Structure	E_b (PW)	E_b (LCAO)
Fe_4+A		
I	-292	-358
II	-269	-311
III	-233	-300
Fe_4+E		
I	-169	-228
II	-148	-202
III	-106	-178

Fe$_5$

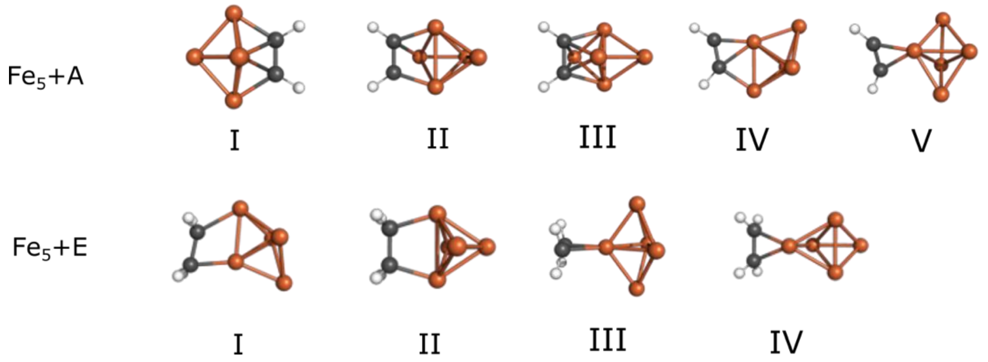

Figure S10. Optimized structures of acetylene and ethylene binded to Fe$_5$.

Table S5. Calculated binding energies (E_b) in kJ/mol of acetylene and ethylene binded to Fe$_5$.

Structure	E_b (PW)	E_b (LCAO)
Fe$_5$+A		
I	-278	-335
II	-260	-316
III	-244	-294
IV	-218	-260
V	-179	-226
Fe$_5$+E		
I	-163	-210
II	-139	-203
III	-128	-177
IV	-123	-159

Fe_6

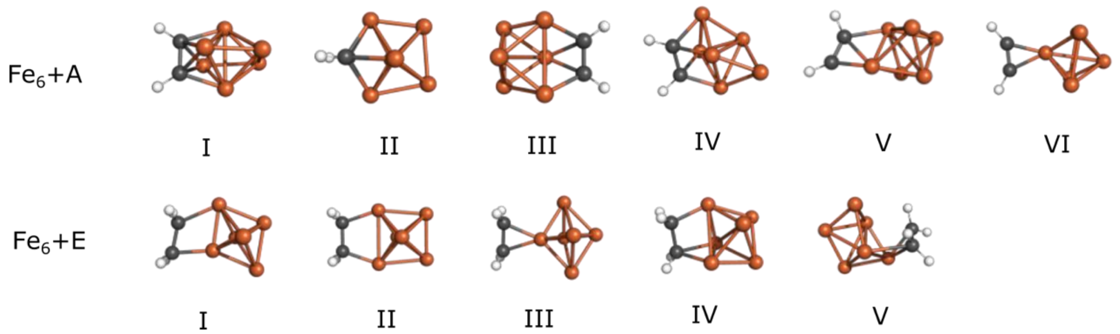

Figure S11. Optimized structures of acetylene and ethylene binded to Fe_6.

Table S6. Calculated binding energies (E_b) in kJ/mol of acetylene and ethylene binded to Fe_6.

Structure	E_b (PW)	E_b (LCAO)
Fe_6+A		
I	-243	-299
II	-239	-297
III	-235	-289
IV	-236	-289
V	-162	-211
VI	-154	-203
Fe_6+E		
I	-139	-195
II	-106	-174
III	-120	-173
IV	-99	-154
V	-86	-128

Fe_7

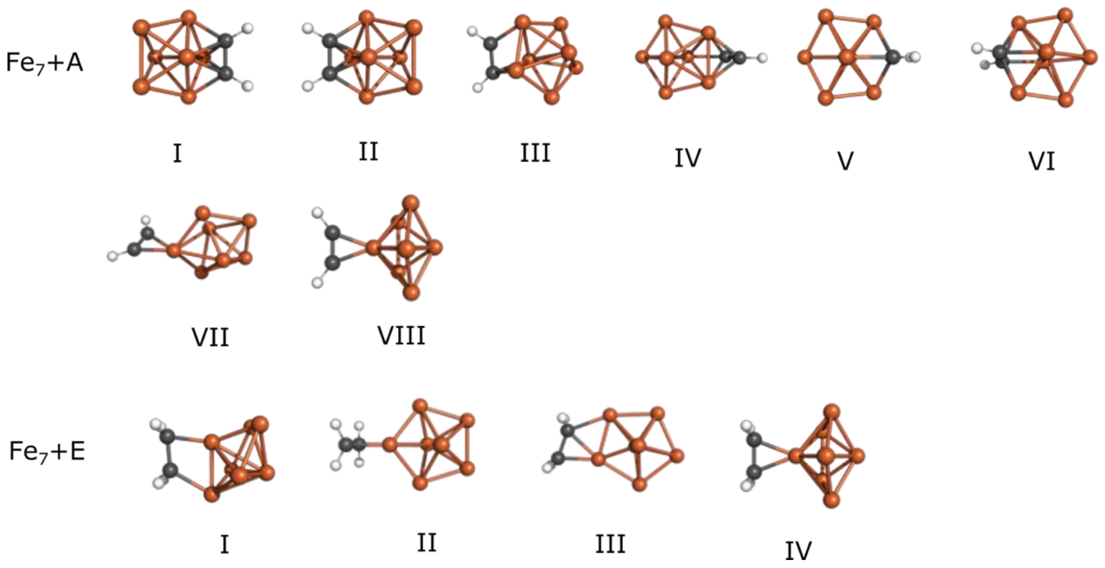

Figure S12. Optimized structures of acetylene and ethylene binded to Fe_7.

Table S7. Calculated binding energies (E_b) in kJ/mol of acetylene and ethylene binded to Fe_7.

Structure	E_b (PW)	E_b (LCAO)
Fe_7+A		
I	-243	-313
II	-243	-312
III	-237	-308
IV	-213	-291
V	-198	-267
VI	-185	-255
VII	-146	-211
VIII	-152	-199
Fe_7+E		
I	-138	-205
II	-108	-173
III	-106	-169
IV	-109	-167

Fe$_8$

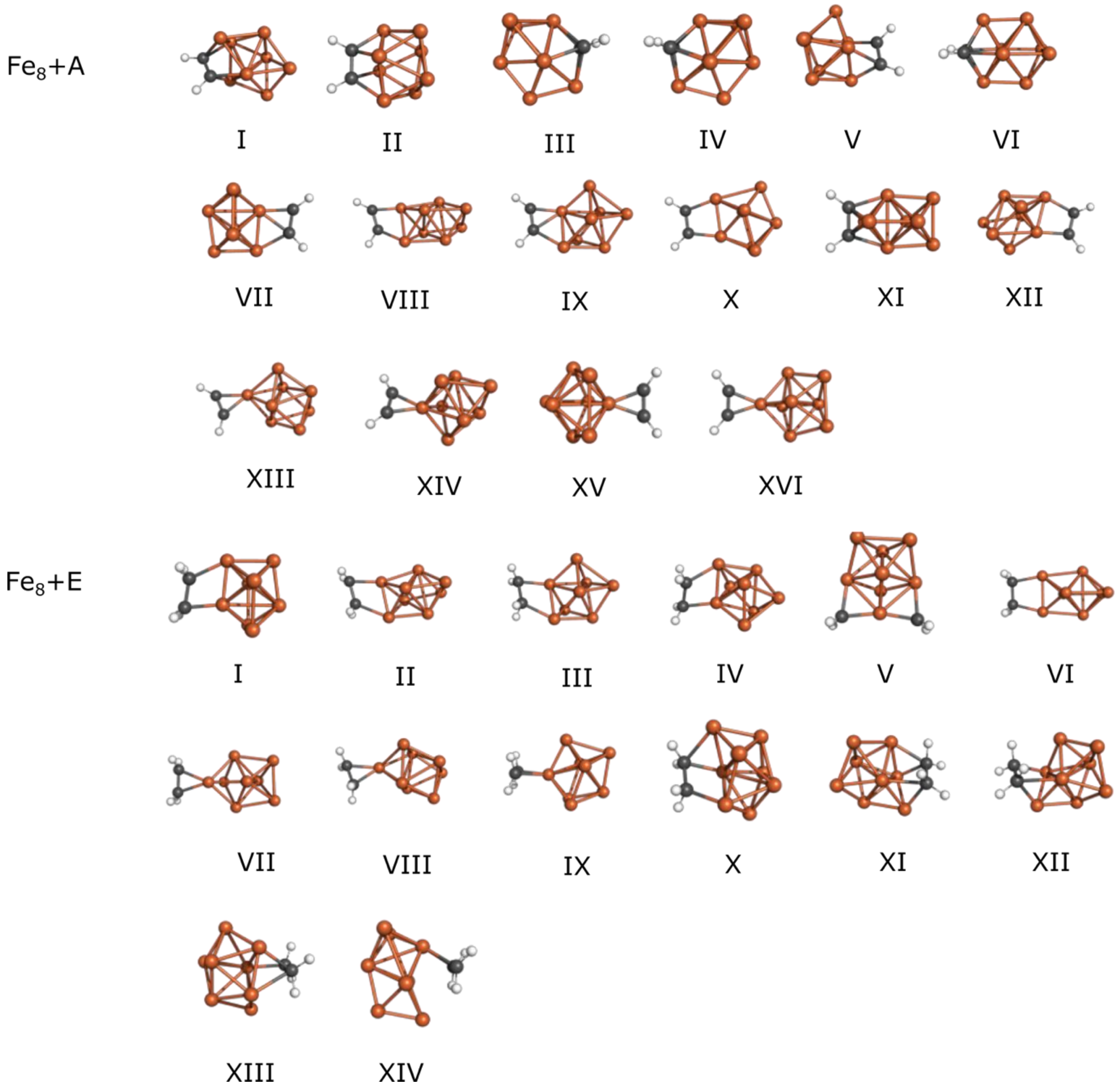

Figure S13. Optimized structures of acetylene and ethylene binded to Fe$_8$.

Table S8. Calculated binding energies (E_b) in kJ/mol of acetylene and ethylene binded to Fe_8.

Structure	E_b (PW)	E_b (LCAO)
Fe_8+A		
I	-218	-311
II	-229	-307
III	-218	-307
IV	-218	-305
V	-214	-302
VI	-206	-301
VII	-174	-244
VIII	-143	-241
IX	-148	-230
X	-148	-229
XI	-175	-225
XII	-139	-222
XIII	-144	-220
XIV	-141	-216
XV	-133	-201
XVI	-122	-188
Fe_8+E		
I	-136	-209
II	-109	-190
III	-108	-188
IV	-111	-186
V	-50	-185
VI	-83	-174
VII	-90	-169
VIII	-99	-167
IX	-94	-167
X	-80	-158
XI	-47	-135
XII	-41	-129
XIII	-30	-124
XIV	-5	-93

Fe_9

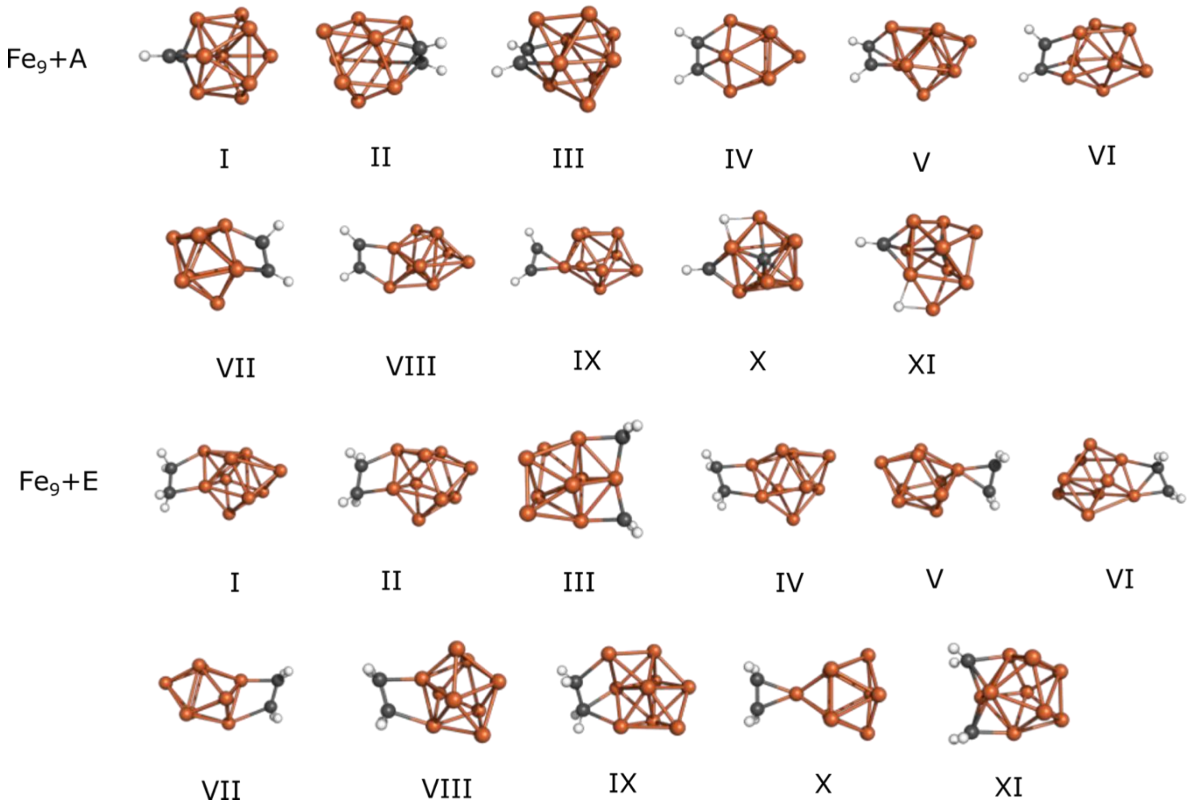

Figure S14. Optimized structures of acetylene and ethylene binded to Fe_9.

Table S9. Calculated binding energies (E_b) in kJ/mol of acetylene and ethylene binded to Fe_9.

Structure	E_b (PW)	E_b (LCAO)
Fe_9+A		
I	-249	-365
II	-237	-351
III	-237	-351
IV	-249	-341
V	-203	-321
VI	-212	-317
VII	-228	-311
VIII	-174	-274
IX	-158	-245
X	-100	-232
XI	-70	-196
Fe_9+E		
I	-130	-226
II	-123	-213
III	-70	-212
IV	-118	-206
V	-112	-206
VI	-111	-205
VII	-105	-202
VIII	-107	-190
IX	-85	-187
X	-103	-175
XI	-52	-171

Fe_{10}

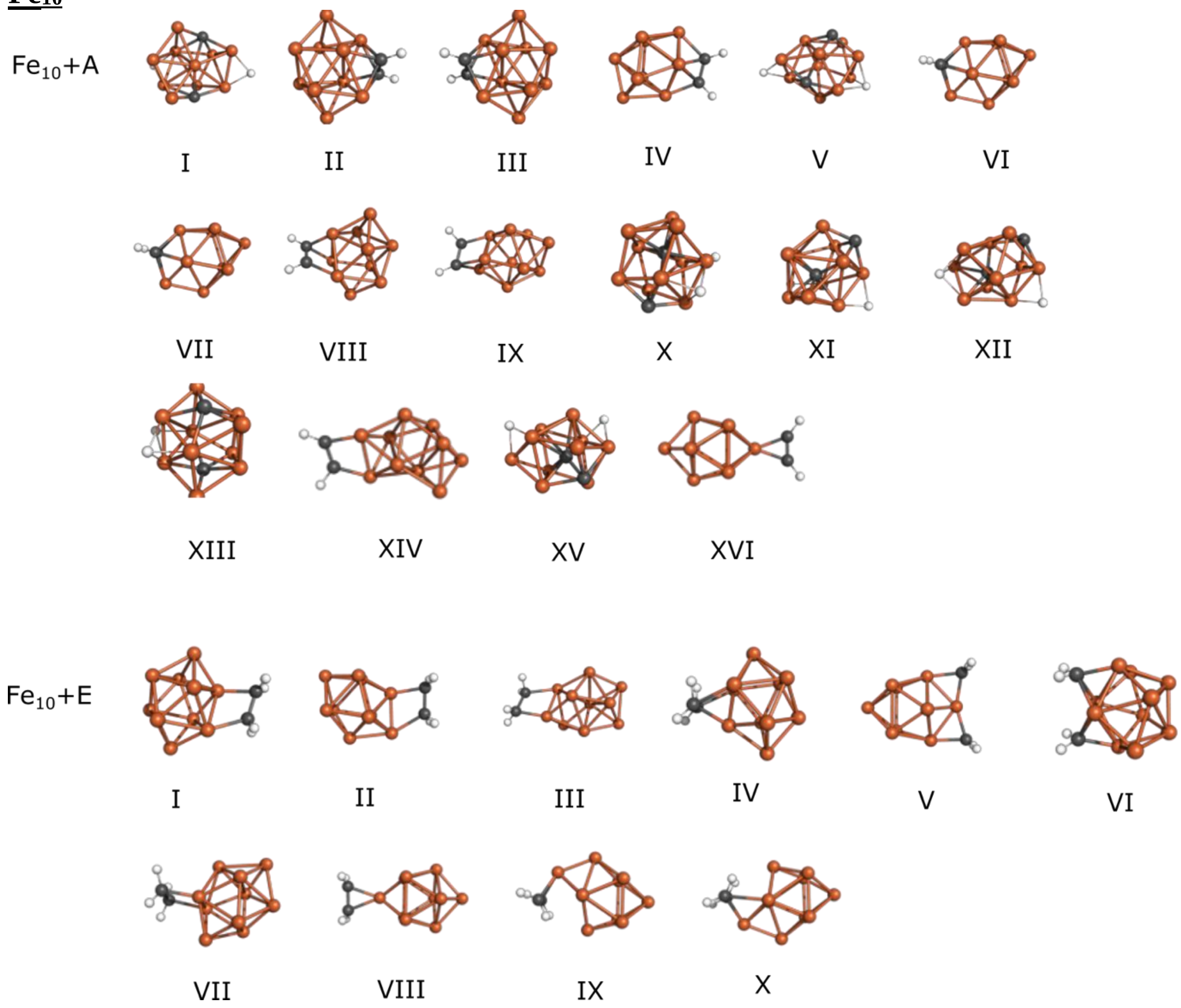

Figure S15. Optimized structures of acetylene and ethylene binded to Fe_{10}.

Table S10. Calculated binding energies (E_b) in kJ/mol of acetylene and ethylene binded to Fe_{10}.

Structure	E_b (PW)	E_b (LCAO)
Fe_{10}+A		
I	-203	-394
II	-249	-380
III	-249	-379
IV	-266	-362
V	-185	-351
VI	-222	-325
VII	-222	-325
VIII	-221	-322
IX	-222	-317
X	-130	-312
XI	-122	-305
XII	-109	-297
XIII	-122	-293
XIV	-161	-279
XV	-50	-237
XVI	-156	-233
Fe_{10}+E		
I	-117	-210
II	-134	-209
III	-94	-204
IV	-107	-199
V	-70	-198
VI	-76	-198
VII	-76	-184
VIII	-111	-179
IX	-30	-118
X	-30	-117

Fe_{13}

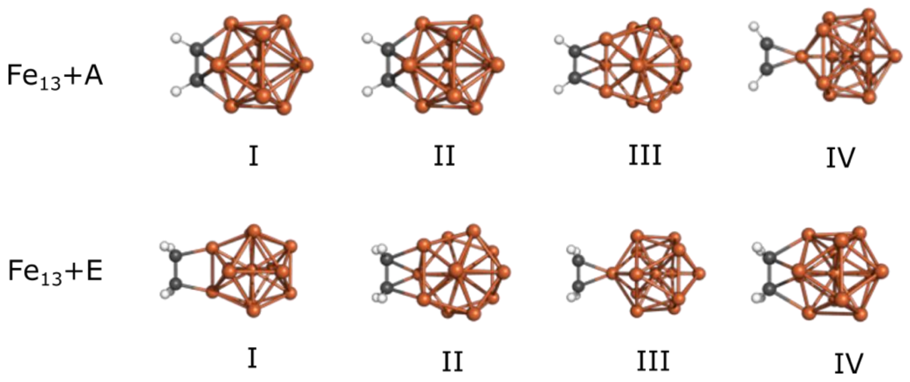

Figure S16. Optimized structures of acetylene and ethylene binded to Fe_{13}.

Table S11. Calculated binding energies (E_b) in kJ/mol of acetylene and ethylene binded to Fe_{13}.

Structure	E_b (PW)	E_b (LCAO)
Fe_{13}+A		
I	-308	-388
II	-308	-388
III	-260	-340
IV	-178	-233
Fe_{13}+E		
I	-135	-209
II	-131	-198
III	-135	-196
IV	-49	-119

Fe_{55}

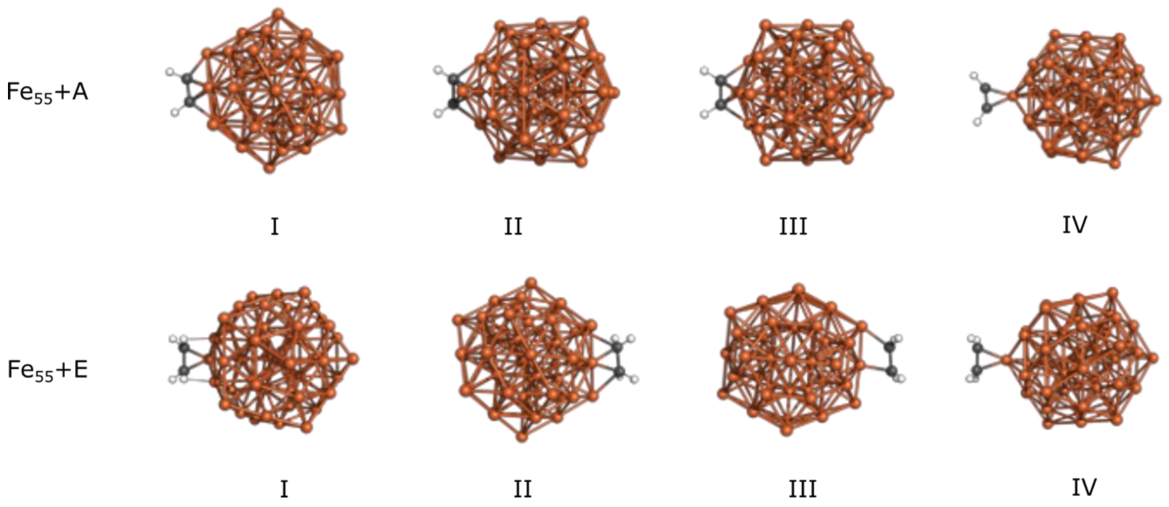

Figure S17. Optimized structures of acetylene and ethylene binded to Fe_{55}.

Table S12. Calculated binding energies (E_b) in kJ/mol of acetylene and ethylene binded to Fe_{55}.

Structure	E_b (PW)	E_b (LCAO)
Fe_{55}+A		
I	-345	-450
II	-322	-421
III	-323	-421
IV	-204	-278
Fe_{55}+E		
I	-166	-256
II	-139	-236
III	-152	-232
IV	-138	-200

6. Atomic positions of the optimized structures

```
Fe3

Fe  -1.91209202  3.07192876  2.61300624
Fe  -2.61905805  2.59749912  0.48561571
Fe  -0.77528887  1.53953798  1.34280869

Fe4

Fe  -0.06126721  1.23807174  0.57009707
Fe  -0.06098546  -0.12477896  -1.35618456
Fe  -0.06148804  -1.11028452  0.78730524
Fe  -1.98630950  0.00023327  -0.00015827

Fe5

Fe  0.00000000  1.25397147  0.57609607
Fe  0.00000000  -0.12432984  -1.37262493
Fe  0.00000000  -1.12224635  0.79638807
Fe  1.90099336  0.00057782  -0.00004772
Fe  -1.90099336  0.00057782  -0.00004772

Fe6

Fe  -0.00278477  1.38179737  1.02945294
Fe  -0.00991316  0.83809698  -1.20093977
Fe  -0.00595040  -0.91344318  1.05018130
Fe  1.79085062  -0.04089230  -0.08008223
Fe  -1.80673614  -0.03837108  -0.07213017
Fe  -0.01272623  -1.45895368  -1.18056200

Fe7

Fe  0.00307731  1.06980196  0.51127488
Fe  -0.06246357  -0.06037220  -1.59796024
Fe  -0.07290112  -1.29766073  0.85495871
Fe  1.81733538  -0.20234263  -0.12044008
Fe  -1.90866101  -0.07602542  -0.07443983
Fe  -1.27568273  -2.05521044  -1.07038633
Fe  1.02721712  -2.13125901  -1.10126855

Fe8

Fe  0.01504489  1.69137621  -0.61881996
Fe  -0.07548596  -0.15925188  -1.99755650
Fe  -0.07690881  -1.71997562  1.00561998
Fe  1.41181601  0.02700727  0.00893404
Fe  -1.47324214  0.12887464  0.06003834
Fe  -1.27973191  -1.89044376  -0.99144606
Fe  1.03926318  -1.97244154  -1.03291328
Fe  0.01340397  0.47232214  1.73082521

Fe9

Fe  -0.08090256  1.00797419  0.69223786
Fe  -0.05973879  -0.95118276  -2.63436831
Fe  1.21740445  -0.99314123  0.81616038
```

```
Fe  1.07579807  0.27177284  -1.09887677
Fe  -1.45859740  0.18963617  -1.07599207
Fe  -1.41759573  -2.10128174  -1.21189217
Fe  1.07624393  -2.23242892  -1.11321590
Fe  -1.30896268  -0.86532434  0.96348363
Fe  -0.26591431  -2.85025349  0.62060320
```

Fe10

```
Fe  -0.08571891  0.84945812  0.59935651
Fe  -0.15320032  -1.15504083  -2.59077606
Fe  1.10994094  -1.09470743  0.90843613
Fe  1.12264058  0.14724175  -1.23094124
Fe  -1.35026737  0.20376634  -1.21481248
Fe  -1.91155948  -1.99642922  -1.06634430
Fe  1.58498237  -2.07465830  -1.08885539
Fe  -1.36396959  -1.03863169  0.92387929
Fe  -0.17259419  -2.91285731  0.43433647
Fe  -0.19759601  -3.32919929  -1.83249574
```

Fe13

```
Fe  2.11636471  2.11624772  2.11720151
Fe  4.13925460  2.11654183  0.86720500
Fe  4.13926761  2.11653907  3.36732334
Fe  0.09469918  2.11746628  0.86673602
Fe  0.09475130  2.11746007  3.36773508
Fe  0.86711555  4.13925826  2.11721307
Fe  3.36759462  4.13870026  2.11723326
Fe  0.86669524  0.09457296  2.11721049
Fe  3.36772698  0.09462451  2.11726324
Fe  2.11719330  0.86717222  4.13964895
Fe  2.11703584  3.36714213  4.13929361
Fe  2.11716851  0.86720695  0.09479339
Fe  2.11700088  3.36713492  0.09512673
```

Fe13S1

```
Fe  10.02764345  10.02745838  10.02744598
Fe  12.09564527  10.02386026   8.75182122
Fe  12.02029533   9.95798175  11.23669581
Fe   8.02604882  10.15265308   8.83457804
Fe   7.99892291  10.06402974  11.32881474
Fe   8.75209500  12.09534694  10.02371847
Fe  11.23676856  12.01959150   9.95868276
Fe   8.83519113   8.02672966  10.15229095
Fe  11.32963372   7.99930545  10.06295684
Fe  10.02408094   8.75199715  12.09578342
Fe   9.95913090  11.23547014  12.02054648
Fe  10.15223434   8.83588541   8.02549530
Fe  10.06297084  11.32945779   7.99882260
S    8.02911048   8.02922732   8.02876074
```

Fe13S7

```
Fe  9.97706727  9.9701821  9.88167706
Fe  12.04100576  10.16454005  8.73018533
Fe  12.17946827  10.04564101  11.15217393
```

Fe	7.71615739	10.01219557	8.81019032
Fe	7.9110886	10.13765713	11.21182631
Fe	8.82622481	12.16909338	9.73194738
Fe	11.29517665	12.13511433	9.8773016
Fe	8.67837405	7.93483478	9.91030111
Fe	11.37875727	7.97720211	9.73625641
Fe	10.16695566	8.67458628	11.82255489
Fe	9.97590642	11.34045395	11.77655527
Fe	9.93693818	8.5073467	7.89151146
Fe	9.81507967	10.99187108	7.82579381
S	7.80793023	8.00325482	7.80241617
S	8.05491801	8.09821135	12.10846895
S	12.28848657	7.90130592	11.79505249
S	6.72012279	11.64971953	10.12007999
S	10.22359751	13.18188776	8.24439479
S	12.15492316	12.12311178	11.92266419
S	12.10219949	8.20239672	7.60034168

Fe13S20

Fe	10.06861187	10.01454410	9.84290086
Fe	12.27944844	9.99753149	8.38404381
Fe	12.36405628	10.00797565	11.16159614
Fe	7.77491002	10.02719823	8.52065066
Fe	7.86537141	10.03399240	11.30102678
Fe	8.69149685	12.27920342	9.88047824
Fe	11.47540185	12.25896001	9.79227575
Fe	8.66335699	7.76995070	9.88955273
Fe	11.44408587	7.75607632	9.80463180
Fe	10.12742862	8.62563143	12.09411731
Fe	10.14674699	11.41132696	12.08650799
Fe	9.98925165	8.61889632	7.59744133
Fe	10.00954252	11.40158144	7.59196637
S	7.84954113	7.88181952	7.77745380
S	7.98355847	7.88988094	12.04975087
S	12.25639566	7.87246477	11.92203285
S	6.62050666	11.36720419	9.94600745
S	10.05006840	13.47798414	8.51559037
S	12.28628326	12.14996147	11.91232134
S	12.12631937	7.85873431	7.63686146
S	10.12726494	13.47920915	11.15274878
S	8.01450864	12.17936339	12.04556786
S	8.86130327	10.02623115	13.34340227
S	11.49340001	10.01355239	13.25552690
S	10.08188610	6.55329269	11.17337226
S	13.54180018	11.31872886	9.72883006
S	12.15826518	12.13100203	7.62762066
S	7.87599246	12.16280291	7.76916290
S	6.60176854	8.71495898	9.95060935
S	10.00771238	6.55040511	8.52356357
S	13.52306351	8.67454504	9.74314538
S	11.28572202	9.99441920	6.34663736
S	8.64526482	10.01891595	6.42169000

Fe55

Fe	12.50093735	12.49432921	12.49995496
Fe	14.44501419	12.49276695	11.29601331

Fe	14.44581979	12.49432746	13.70257887
Fe	10.55432197	12.49541468	11.29844329
Fe	10.55474243	12.49484429	13.70191232
Fe	11.29957312	14.44056481	12.49966019
Fe	13.70334822	14.43897660	12.49928289
Fe	11.30012157	10.54791041	12.50038182
Fe	13.70593553	10.54961406	12.50089924
Fe	12.49971693	11.29161990	14.44568867
Fe	12.50045937	13.69642324	14.44510852
Fe	12.49792326	11.29032154	10.55499058
Fe	12.50050366	13.69541276	10.55422427
Fe	16.50274807	12.49275804	10.01964902
Fe	16.52420036	12.49175710	12.49726217
Fe	16.50510044	12.49661943	14.97603733
Fe	8.49202220	12.50207648	10.02869720
Fe	8.47343384	12.49754946	12.50032295
Fe	8.49275242	12.49697215	14.97093427
Fe	10.03046182	16.50284112	12.49931607
Fe	12.50223260	16.52087832	12.49867060
Fe	14.97368358	16.49539752	12.49918226
Fe	10.03964970	8.48238793	12.49896803
Fe	12.51185720	8.46717914	12.50485987
Fe	14.98856184	8.49532032	12.50167354
Fe	12.49484272	10.01884876	16.50732572
Fe	12.49501890	12.49234828	16.52540615
Fe	12.50174872	14.96778499	16.50522477
Fe	12.49022778	10.01586411	8.49505319
Fe	12.49342903	12.48912551	8.47481473
Fe	12.50384526	14.96563491	8.49327391
Fe	14.51119331	11.25081284	15.75902248
Fe	14.51291774	13.73648906	15.75900163
Fe	14.50799472	11.24518599	9.24111371
Fe	14.51235666	13.73299859	9.23875393
Fe	10.48315160	11.25038190	15.75610635
Fe	10.48530498	13.74025259	15.75606038
Fe	10.47961192	11.24985454	9.24536926
Fe	10.48611457	13.73963762	9.24326362
Fe	15.75847780	14.50499287	11.25303948
Fe	15.75944745	14.50655423	13.74415587
Fe	9.24463030	14.51268486	11.25627699
Fe	9.24470478	14.51167043	13.74341283
Fe	15.75999017	10.47975409	11.25119246
Fe	15.76271731	10.48300733	13.74664466
Fe	9.24400903	10.48207685	11.25972882
Fe	9.24503522	10.47985865	13.74228815
Fe	11.25889088	15.75289318	14.51354513
Fe	13.74613556	15.75099745	14.51470392
Fe	11.25825953	9.23608023	14.51382726
Fe	13.74877483	9.24010744	14.51712892
Fe	11.25962302	15.75186791	10.48464117
Fe	13.74673243	15.74974176	10.48320952
Fe	11.25515788	9.23405454	10.48815890
Fe	13.74236364	9.23648088	10.48882365

Fe55S1

Fe	13.72372843	12.27244838	12.49518538
Fe	15.67097855	12.27737437	11.2938653

Fe 15.66368448 12.28016497 13.69083994
Fe 11.76834361 12.25922232 11.27322354
Fe 11.76605132 12.25307218 13.66762305
Fe 12.4989946 14.19803479 12.46457479
Fe 14.90259279 14.22804506 12.49663331
Fe 12.53009962 10.31485174 12.49458604
Fe 14.93102253 10.32859959 12.49575006
Fe 13.72009038 11.07687991 14.44057854
Fe 13.71299661 13.47869948 14.44169931
Fe 13.72866372 11.0697541 10.55320399
Fe 13.73076123 13.47167379 10.54146062
Fe 17.74193076 12.28768665 10.03412714
Fe 17.75968699 12.28809004 12.49020366
Fe 17.72551181 12.28248998 14.94251577
Fe 9.78485466 12.19921234 9.95432361
Fe 9.66038094 12.17738573 12.51556919
Fe 9.6798896 12.22117901 14.94047865
Fe 11.20484169 16.27133319 12.431652
Fe 13.67754447 16.29400499 12.49150125
Fe 16.14531464 16.30154654 12.53013569
Fe 11.2854356 8.235502 12.52115961
Fe 13.74119476 8.23452394 12.51201127
Fe 16.2023986 8.26571344 12.50642389
Fe 13.73257091 9.8370229 16.51742506
Fe 13.71464027 12.29232369 16.52653529
Fe 13.68932533 14.74632845 16.49612773
Fe 13.75751605 9.7815757 8.48090258
Fe 13.75703769 12.26498951 8.46532914
Fe 13.78104561 14.74821186 8.4804134
Fe 15.7401276 11.05061786 15.7514755
Fe 15.72147219 13.53393777 15.74614873
Fe 15.74909615 11.03418491 9.24336122
Fe 15.76297529 13.52818414 9.24154558
Fe 11.70663047 11.03657641 15.74311652
Fe 11.67841539 13.50680904 15.70986157
Fe 11.76127208 10.98936761 9.20777162
Fe 11.74969832 13.49210659 9.20366339
Fe 16.96439919 14.29890074 11.27124362
Fe 16.95548361 14.30695709 13.74394838
Fe 10.47009069 14.3347468 11.18140923
Fe 10.45073373 14.23889988 13.67995176
Fe 16.98081444 10.264779 11.25161372
Fe 16.98891078 10.26352015 13.73537436
Fe 10.49830566 10.21346704 11.23199101
Fe 10.49004483 10.21535196 13.74826641
Fe 12.42635665 15.49998347 14.47747481
Fe 14.92304692 15.54722367 14.52308648
Fe 12.50818874 9.00381025 14.53225837
Fe 14.98026237 9.03421855 14.51758247
Fe 12.50350233 15.52356669 10.45606574
Fe 14.98145954 15.54546229 10.48632713
Fe 12.50488244 9.00026164 10.48661097
Fe 14.97386823 9.01193835 10.48850696
S 8.3895677 13.4631063 11.17237298

Fe55S5

Fe 13.74811821 12.26844807 12.48566873

```
Fe  15.66518707  12.26776769  11.28824584
Fe  15.67977205  12.29514689  13.67707162
Fe  11.75949649  12.28652605  11.29285703
Fe  11.74644309  12.27499222  13.69469625
Fe  12.49753792  14.24741082  12.4473101
Fe  14.92177297  14.24073092  12.45998383
Fe  12.52453414  10.3487366  12.52557586
Fe  14.92259386  10.34155807  12.49905045
Fe  13.74359071  11.09270595  14.45620811
Fe  13.70152657  13.48265577  14.41823371
Fe  13.73334678  11.04102496  10.54419375
Fe  13.73479328  13.46361171  10.53381342
Fe  17.72253102  12.22537663  10.00642518
Fe  17.76979911  12.30000886  12.47223797
Fe  17.7635691  12.37429901  14.9046313
Fe  9.80377357  12.15794203  9.94925488
Fe  9.60887705  12.26118907  12.4603393
Fe  9.70147181  12.4505322  14.91534355
Fe  11.29123028  16.27826819  12.35662801
Fe  13.74926052  16.30723678  12.469781
Fe  16.14244249  16.35690321  12.45808338
Fe  11.21937114  8.29472058  12.65525603
Fe  13.70584596  8.27126771  12.537626
Fe  16.17390257  8.27318053  12.49313485
Fe  13.84445527  9.81214772  16.50405688
Fe  13.78578237  12.2581154  16.52786325
Fe  13.65775063  14.66595948  16.57346478
Fe  13.72631185  9.71531724  8.50364575
Fe  13.73967862  12.24659559  8.47846667
Fe  13.81841608  14.79462588  8.49484074
Fe  15.82243372  11.06479008  15.72482262
Fe  15.71336603  13.55848592  15.69757132
Fe  15.72398891  10.97979238  9.23425773
Fe  15.74867245  13.47736968  9.23422385
Fe  11.75249288  11.02877604  15.77126147
Fe  11.77925888  13.44525089  15.76674004
Fe  11.78613238  10.97517733  9.23830988
Fe  11.77982803  13.49099296  9.19471473
Fe  16.98059313  14.27338437  11.25494782
Fe  16.92322661  14.35646098  13.71533427
Fe  10.42572239  14.28355245  11.09579624
Fe  10.45720282  14.26243469  13.56597182
Fe  16.97184231  10.25058911  11.24939152
Fe  17.01972876  10.29970832  13.71310735
Fe  10.52385845  10.24018755  11.28402352
Fe  10.50860843  10.27852586  13.82154982
Fe  12.34934571  15.46241161  14.54979998
Fe  14.85365993  15.5246959  14.48513399
Fe  12.52479451  9.0242094  14.57015338
Fe  15.03402173  9.03886295  14.51622033
Fe  12.61364116  15.53575321  10.44625473
Fe  15.05031064  15.55159484  10.45638247
Fe  12.46309231  8.99025328  10.53406386
Fe  14.95828748  8.96852423  10.48750354
S  8.38469258  13.46444692  11.0742322
S  10.56619627  16.32297284  10.23400612
S  12.44999004  17.56250077  13.78835105
S  9.89579647  14.56069381  15.7044285
```

S 8.43594907 10.96174027 13.86670742

Fe3a

Fe -3.40707607 1.89429544 0.38150319
Fe -1.14185359 1.84991926 0.33581765
Fe -2.28090195 0.31868315 1.51106611
C -3.02302903 0.17578702 -0.36807775
C -1.60423012 0.13696122 -0.38558474
H -3.71173255 -0.56780559 -0.82625043
H -0.96714324 -0.64541465 -0.85451504

Fe4a

Fe -0.27409988 -0.33672498 0.88127354
Fe -0.59314465 1.12168240 -0.83932309
Fe 1.21113463 -0.49654023 -0.87079731
Fe -0.99612620 -1.09817801 -1.15066406
C -2.20498728 0.37981331 0.02477508
C -2.11757462 -0.94333710 0.47524573
H -3.12297128 1.00418509 0.13873164
H -2.92220939 -1.51374142 0.97807889

Fe5a

Fe -1.78993470 2.08350328 0.14120627
Fe -1.07275538 0.48499765 -1.41508288
Fe -1.27824738 0.17090628 1.39260529
Fe 0.41041105 1.16117773 0.19667466
Fe -2.94612915 0.02258672 -0.17475710
C -0.04628182 -0.79144005 -0.04819436
C -1.36912365 -1.23718141 -0.20318229
H 0.79906121 -1.51107322 -0.06001776
H -1.59125309 -2.31551117 -0.34423013

Fe6a

Fe 0.44517069 1.41710672 1.39379410
Fe 0.37185137 0.45347325 -0.82653335
Fe -0.62145514 -0.75668385 1.38802837
Fe 1.59195358 -0.58115582 0.81631070
Fe -1.56833596 1.15281183 0.34731657
Fe -0.30552645 -1.68271980 -0.63822093
C -2.08483157 -0.78700483 -0.06695639
C -1.56234239 -0.14712861 -1.23411575
H -3.10131339 -1.23685931 -0.02300493
H -2.13622082 -0.05051507 -2.18243746

Fe7a

Fe -1.91342274 1.87999115 -0.04626426
Fe -1.91239568 0.49967170 -1.92128676
Fe -1.41739406 -0.24667644 0.65884099
Fe 0.19070492 0.92810257 -0.64142968
Fe -3.49328901 -0.01159547 -0.29109473
Fe -2.10247809 -1.81513065 -0.99238751
Fe -0.12055596 -1.02317039 -1.74750111

C 0.52586990 -0.86858652 0.23262210
C -0.39605015 -1.97196363 0.09094360
H 1.42686906 -1.01652994 0.87031169
H -0.16382124 -2.92053158 0.62594688

Fe8a

Fe -0.58364599 1.44554901 -0.01790371
Fe -0.29250568 -0.25430122 -1.47854463
Fe 0.78466417 -1.64003768 1.29764046
Fe 1.51563082 0.47062504 0.12533396
Fe -1.48321735 -0.58008345 0.96599735
Fe -0.76403068 -2.31425368 -0.29072779
Fe 1.47220247 -1.58458048 -0.89994192
Fe 0.23946092 0.46332503 2.04360425
C -2.18098876 0.38506083 -0.67485800
C -2.12170784 -1.00910826 -1.04046886
H -3.09615712 0.95817383 -0.95821095
H -2.98195392 -1.43702152 -1.60845154

Fe9a

Fe -1.05143244 0.08127818 1.24244322
Fe -0.69155355 -1.45554420 -2.12183934
Fe 1.19200831 -0.87783803 1.08649446
Fe 0.05922299 0.21576182 -0.75666741
Fe -2.07044587 -1.28374422 -0.27906077
Fe -0.71673613 -3.23058042 -0.44976226
Fe 1.34282384 -1.86651068 -0.94758587
Fe -0.80119545 -2.26682940 1.61173529
Fe 1.20367066 -3.24065073 0.93137232
C -1.93775565 0.82881521 -0.47189923
C -1.83729564 0.21688752 -1.74051369
H -2.55802589 1.74557251 -0.35216821
H -2.37751187 0.66113173 -2.60684522

Fe10a

Fe 0.45716164 0.25385357 1.13337260
Fe 1.26356360 -1.74096003 -1.93343708
Fe 0.36470084 -2.08826502 1.63209375
Fe 2.06760990 -1.05351835 0.16638729
Fe 0.05738181 0.10761799 -1.11132749
Fe -1.16229326 -1.81265607 -1.68142552
Fe 1.39213097 -3.28896706 -0.04727468
Fe -1.45003433 -1.03961504 0.47324123
Fe -1.07839312 -3.33429693 0.29291224
Fe -0.04777140 -3.79412384 -1.73118213
C 1.81119802 0.90403822 -0.39268267
C 2.07641300 0.11799167 -1.54212630
H 2.29173915 1.89237543 -0.23783957
H 2.77621495 0.46864428 -2.33220643

Fe13a

Fe 1.33408333 1.62596274 2.25658842
Fe 2.74884390 1.35007427 0.44904719
Fe 3.65153761 1.78644065 2.71713188

Fe -0.91211891 1.45528855 1.73661787
Fe -0.17274440 1.89607870 4.06292214
Fe 0.08056460 3.71083816 2.45101260
Fe 2.53619654 3.64134132 1.58313460
Fe 0.21713646 -0.34372933 3.16533784
Fe 2.67263537 -0.41444387 2.30477668
Fe 2.04865526 0.77138872 4.36096281
Fe 1.97096213 3.20218542 3.92526729
Fe 0.80010868 -0.25519059 0.88559023
Fe 0.69110082 2.87850062 0.33228342
C -0.30474253 1.13511025 -0.17925629
C 1.06356887 1.09921807 -0.66232045
H -1.13366251 0.96979533 -0.90800085
H 1.24267170 0.91734539 -1.74901704

Fe55a

Fe 13.71448619 12.27325743 12.49290359
Fe 15.66282431 12.28918866 11.29866653
Fe 15.65833772 12.29007219 13.69488274
Fe 11.78007570 12.27597152 11.27204223
Fe 11.76713812 12.28371999 13.69282808
Fe 12.48241114 14.21889036 12.48568849
Fe 14.89199689 14.22899702 12.49720885
Fe 12.51209943 10.32149125 12.49505537
Fe 14.92261507 10.33812802 12.50008226
Fe 13.71914870 11.07789052 14.43849622
Fe 13.70613798 13.48154381 14.44215567
Fe 13.73224923 11.06968598 10.55271475
Fe 13.71842018 13.48120469 10.54324119
Fe 17.73128095 12.30342073 10.03001384
Fe 17.75022211 12.29256312 12.49521458
Fe 17.72836835 12.29975095 14.95851279
Fe 9.78726456 12.20411885 9.91444510
Fe 9.71910937 12.35092053 12.46511289
Fe 9.74267693 12.24112958 15.01500972
Fe 11.22290254 16.23045308 12.48091447
Fe 13.71068952 16.29432282 12.49863749
Fe 16.17016820 16.30814364 12.51135345
Fe 11.21823615 8.27796834 12.50896533
Fe 13.70434234 8.25226277 12.51045587
Fe 16.19149594 8.27865842 12.50731760
Fe 13.74567593 9.79249462 16.49666642
Fe 13.73940984 12.26648599 16.53245152
Fe 13.70871991 14.74338010 16.51275199
Fe 13.77586608 9.76752950 8.50371729
Fe 13.76734793 12.25731737 8.46182402
Fe 13.73193709 14.75392973 8.48163425
Fe 15.74104685 11.04520955 15.73910559
Fe 15.72572462 13.53441189 15.74878391
Fe 15.75663891 11.03738250 9.25669991
Fe 15.74111165 13.53827642 9.24507641
Fe 11.73199649 11.01856905 15.74857230
Fe 11.71634623 13.50049327 15.78257966
Fe 11.76485979 10.99139610 9.21796490
Fe 11.74671207 13.49216273 9.17646056
Fe 16.95783408 14.31083564 11.26387768
Fe 16.95620976 14.30899032 13.73801331

```
Fe  10.45366828  14.27560290  11.11364283
Fe  10.42654253  14.27985136  13.82506207
Fe  16.97558876  10.27388351  11.25799860
Fe  16.97609565  10.27525489  13.74139003
Fe  10.46182312  10.26070917  11.25374106
Fe  10.44616614  10.27656875  13.71723995
Fe  12.46448284  15.52133879  14.51848327
Fe  14.93939296  15.54870191  14.51261877
Fe  12.45901440  9.02259201  14.51858559
Fe  14.95924406  9.01597153  14.50934191
Fe  12.48229728  15.53013616  10.45710117
Fe  14.95102592  15.55402494  10.49175458
Fe  12.47623431  9.00720482  10.48676953
Fe  14.96774441  9.00394400  10.50024256
C  9.39383412  15.47408705  12.45682154
C  8.86577066  14.14660706  12.45217216
H  8.67582673  16.33021737  12.44818034
H  7.75947373  13.99838130  12.44217869
```

Fe3e

```
Fe  -4.20848688  0.54329729  0.75851431
Fe  -2.09676108  0.57526991  -0.07070838
Fe  -2.46172802  0.99371191  2.11913014
C  -4.25260032  -1.08172806  -0.40042419
C  -2.86977465  -1.04517015  -0.95675441
H  -5.06421339  -0.94874606  -1.14873571
H  -4.47548734  -1.93412244  0.27884416
H  -2.81122195  -0.88540095  -2.05589759
H  -2.20576682  -1.88254204  -0.64424861
```

Fe4e

```
Fe  -2.02717539  1.98261176  0.93922165
Fe  -2.40149266  0.13543336  -0.37596400
Fe  -0.22513417  0.95429933  0.01628369
Fe  -1.38622076  -0.20831745  1.66911084
C  -3.21653865  1.98156556  -0.99075663
C  -2.63531744  3.23352126  -0.45624587
H  -2.87298945  1.74038824  -2.02481013
H  -4.32036860  1.88508787  -0.90743459
H  -1.85715408  3.72974674  -1.06361942
H  -3.34200190  3.94754885  0.01543565
```

Fe5e

```
Fe  -2.42355888  -1.18788796  -1.00528258
Fe  -0.84807432  -0.04489208  -2.34557526
Fe  -0.58820723  -2.39886346  -1.86326048
Fe  -0.25198129  -0.78117535  -0.20870004
Fe  -2.32094209  -1.63356919  -3.26083760
C  -3.42122025  -0.92417616  0.68249554
C  -2.00289159  -0.70290764  1.02188894
H  -3.90128064  -1.83887017  1.07891916
H  -4.08643256  -0.03999246  0.68273674
H  -1.74316209  0.33750658  1.32767911
H  -1.55702379  -1.45707287  1.71195241
```

Fe6e

```
Fe  -1.45476398  -1.40776134  0.10027194
Fe  -1.44427087  0.33496911  -1.29915274
Fe  0.72295732  -2.05612478  0.31157499
Fe  -0.09761900  0.26151729  0.89296124
Fe  -0.52716161  -1.98861225  -1.90848571
Fe  0.79228803  -0.29720205  -1.13373900
C  -3.35949074  -1.55904624  0.49816410
C  -3.26601326  -0.39857525  -0.42147217
H  -3.83431607  -2.47837329  0.10394088
H  -3.62331534  -1.34558298  1.55198903
H  -3.77426753  -0.54513245  -1.40182847
H  -3.54638658  0.57542715  0.03951757
```

Fe7e

```
Fe  -1.74691854  -1.73274940  1.24280900
Fe  -1.45527389  -1.27881362  -0.98046280
Fe  0.27875856  -2.98208734  0.28932126
Fe  0.25958901  -0.73370161  0.72032270
Fe  -1.88783325  -3.57248690  -0.13095367
Fe  -0.55258545  -3.15922319  -1.98241205
Fe  0.83396549  -1.40474174  -1.42967742
C  -3.06023443  -0.43537885  0.24524093
C  -2.98332751  -0.08331295  -1.19516150
H  -3.97711169  -1.00869101  0.51995371
H  -2.89935097  0.42931034  0.93056020
H  -2.69331442  0.95641890  -1.44421315
H  -3.77772860  -0.48754430  -1.85286948
```

Fe8e

```
Fe  -1.84777735  0.40835580  0.64301559
Fe  -1.44092313  -1.45713736  -0.53360220
Fe  1.15365620  -1.33732589  0.62216809
Fe  0.08217482  0.50197016  -0.70158105
Fe  -0.99339268  -1.37808880  1.91210812
Fe  -0.29119460  -3.07418578  0.49411372
Fe  0.54641401  -1.61016798  -1.54144726
Fe  0.28305882  0.53049298  1.60690483
C  -3.35261503  -0.46449788  -0.57937044
C  -3.14985639  -1.69138339  -1.39715323
H  -3.55285805  0.45339813  -1.17984698
H  -4.10518703  -0.57793852  0.23513715
H  -3.12352443  -1.56094714  -2.49772198
H  -3.69683863  -2.60300607  -1.08386420
```

Fe9e

```
Fe  -2.72586445  -0.44570324  -1.43114618
Fe  1.30346615  -1.25643319  -3.00429579
Fe  -1.37399391  -1.30488011  0.05788752
Fe  -0.18052449  0.32500176  -1.16073732
Fe  -0.94720000  -0.93569630  -2.88119176
Fe  0.08712953  -3.17211340  -2.63348091
Fe  0.91068642  -1.66104048  -0.82564152
Fe  -2.11836223  -2.72426494  -1.92542424
```

```
Fe  -0.53898832  -3.39307832  -0.38592912
C  -3.39870602  -0.54284935  0.55861426
C  -2.52821085  -1.10266924  1.61741446
H  -4.34363919  -1.10893117  0.38671390
H  -3.60218574  0.54985315  0.66161376
H  -2.10510444  -0.39210148  2.35687470
H  -2.84254935  -2.06211057  2.07467737
```

Fe10e

```
Fe  -2.78044899  -0.62509003  -1.43443492
Fe  0.61309016  -1.58442466  -3.03859439
Fe  -1.40219075  -1.51775683  0.08814218
Fe  -0.50908354  -0.05767350  -1.70836576
Fe  -1.65237412  -1.50882985  -3.29454338
Fe  -0.81248681  -3.60075567  -2.93812411
Fe  0.79209845  -1.58113213  -0.56934838
Fe  -2.56007045  -2.96668158  -1.56609489
Fe  -0.63853813  -3.60368693  -0.46581337
Fe  1.20550121  -3.45886421  -1.85449344
C  -3.14599158  -0.27585650  0.63698300
C  -2.25947670  -0.81804387  1.68683901
H  -4.18529251  -0.67579625  0.65573180
H  -3.14771403  0.83554569  0.56065679
H  -1.61458984  -0.10002452  2.23275823
H  -2.66664212  -1.61953677  2.33576961
```

Fe13e

```
Fe  1.47953061  1.39996889  1.51911751
Fe  3.53304638  2.17076097  0.58520805
Fe  3.12813335  2.19949834  3.04683372
Fe  -0.24910586  0.58005026  0.06038868
Fe  -0.62190078  0.59863020  2.36487985
Fe  -0.41160043  2.82261202  1.20636819
Fe  1.89988974  3.75555067  1.57939603
Fe  1.14660944  -0.96345172  1.47285942
Fe  3.42842680  0.04872722  1.83913134
Fe  1.63985831  0.26204813  3.55737718
Fe  0.67838172  2.57045429  3.39295349
Fe  2.27076543  0.22488548  -0.35597884
Fe  1.32466337  2.53818329  -0.51932569
C  -2.10585174  -0.17731130  0.18969693
C  -2.35497770  -0.13534536  1.66561220
H  -2.07269789  -1.19985659  -0.24987420
H  -2.76160128  0.49056946  -0.41375038
H  -2.49738804  -1.12730429  2.15148173
H  -3.14850139  0.57859180  1.98245055
```

Fe55e

```
Fe  12.48550308  13.66270872  12.55005893
Fe  14.43336001  13.56468934  11.36105326
Fe  14.41984849  13.61377432  13.76197700
Fe  10.54639426  13.66067318  11.33524400
Fe  10.52445455  13.77235653  13.73338626
Fe  11.33487542  15.63590576  12.47606916
Fe  13.74407901  15.55702907  12.49538375
```

```
Fe  11.21486122  11.73773300  12.61587274
Fe  13.59600395  11.66092251  12.60949177
Fe  12.40245674  12.52775397  14.53633351
Fe  12.52070476  14.92983463  14.45114792
Fe  12.46274596  12.40085491  10.63612206
Fe  12.52147409  14.80098230  10.56904746
Fe  16.51401369  13.47671904  10.12263670
Fe  16.50795360  13.49634443  12.58236181
Fe  16.46833913  13.52267349  15.03379028
Fe  8.49669791  13.62986388  10.03312827
Fe  8.45082634  13.79986388  12.49821307
Fe  8.46119343  13.93151391  14.99629126
Fe  10.10837258  17.72450133  12.41142865
Fe  12.60378687  17.67322511  12.42494022
Fe  15.07851956  17.57606000  12.43571709
Fe  9.93631940  9.77168147  12.70631810
Fe  12.31474022  9.56793997  12.62488875
Fe  14.76122990  9.54337374  12.68687634
Fe  12.32742898  11.35783100  16.65387820
Fe  12.48279342  13.81540212  16.58141083
Fe  12.61299044  16.26844597  16.46405487
Fe  12.49731644  11.10812479  8.59011291
Fe  12.52947090  13.55966782  8.51446981
Fe  12.54552296  16.02497290  8.47881871
Fe  14.41301729  12.41285428  15.85057680
Fe  14.55013221  14.90926326  15.76393010
Fe  14.50792130  12.28349646  9.33990117
Fe  14.55093260  14.76937828  9.28776536
Fe  10.39170952  12.66424711  15.85481720
Fe  10.51546263  15.12736473  15.73982331
Fe  10.47798351  12.37518567  9.29133639
Fe  10.51689791  14.84558834  9.24354896
Fe  15.81654486  15.53318736  11.27379271
Fe  15.80553537  15.56846658  13.74112432
Fe  9.29070166  15.70581812  11.21247591
Fe  9.27527107  15.83475065  13.70662978
Fe  15.67114956  11.50542508  11.40316747
Fe  15.62305157  11.51983258  13.88514902
Fe  9.17904077  11.68832488  11.32582916
Fe  9.10742477  11.85630413  13.80585099
Fe  11.36047609  17.02858373  14.43744742
Fe  13.85825981  16.93384055  14.46388571
Fe  10.98050720  10.55922857  14.72530300
Fe  13.48165496  10.41785364  14.64691511
Fe  11.32789214  16.89027303  10.43159171
Fe  13.82313469  16.81903378  10.44970966
Fe  11.16397487  10.40533190  10.60414879
Fe  13.65066541  10.30899393  10.62921918
C  10.88560322  8.03970070  13.23861090
C  10.11534578  8.59461280  14.35999792
H  10.48741954  7.17630742  12.66761607
H  11.99470473  7.86903799  13.46708800
H  9.12941310  8.15921327  14.62271620
H  10.71556298  8.76564900  15.32186666
```

fe13s1a_0

```
Fe 10.00069756 10.05673144 9.74619376
```

Fe 11.90003072 10.02708701 8.47538942
Fe 12.05399766 10.02145839 10.99349223
Fe 8.10168737 10.01452894 8.45231346
Fe 8.04556242 10.04045910 11.01054906
Fe 8.71711013 12.09731456 9.93972569
Fe 11.37139373 12.06990665 9.89993663
Fe 8.85994566 7.94502019 10.07980418
Fe 11.31028047 8.02623321 9.80419811
Fe 10.11064294 8.90760750 11.90049247
Fe 10.08657267 11.31602312 11.81444008
Fe 9.86906698 8.51923653 7.86613312
Fe 10.00487893 11.73150056 8.03117594
S 7.72149738 7.75104786 8.17603147
C 9.30245416 10.25546594 6.83403393
C 10.73646599 10.12751611 6.85132156
H 8.74145016 10.29342865 5.87189809
H 11.28023279 10.15394543 5.87690544

fe13s1a_1

Fe 10.20140768 9.95893863 9.96829662
Fe 12.13359167 10.43826771 8.65169712
Fe 12.15828410 9.92702305 11.15748001
Fe 8.23879112 9.98927973 8.70365830
Fe 8.14740615 10.07177032 11.19078138
Fe 8.94029800 12.03142784 9.97468611
Fe 11.34104543 12.10997215 10.10682701
Fe 8.82292325 7.96680760 10.15701791
Fe 11.17774777 7.78209778 10.20131641
Fe 10.12112128 8.72086902 12.09962484
Fe 10.18365445 11.13703943 12.02130634
Fe 10.31272390 8.71287219 8.02315231
Fe 10.07535054 11.33845541 7.95706022
S 8.18739065 7.84825580 7.98623038
C 12.24695418 8.37827291 8.47821438
C 12.93173214 8.79566424 9.68233040
H 12.80395811 7.73778430 7.75162934
H 13.98075031 8.44960741 9.85226840

fe13s1a_2

Fe 10.19365633 10.14863094 10.08783823
Fe 12.20178433 10.19886227 8.93259876
Fe 12.12476082 9.76242336 11.44072785
Fe 8.15889925 10.17723110 8.83889389
Fe 8.12584944 10.11576603 11.32049015
Fe 8.77337613 12.14692067 10.12795517
Fe 11.12218729 12.32937611 9.81767978
Fe 8.97210851 8.10214438 10.13702958
Fe 11.40524334 8.01860520 10.08044172
Fe 10.04933545 8.88055414 12.18101120
Fe 10.20235051 11.36470475 12.01524260
Fe 10.23273398 8.92484520 8.11370522
Fe 10.09135752 11.36914909 7.96602388
S 8.11703858 8.04783523 8.02069371
C 12.86044926 11.41249162 10.44501465
C 12.09946397 11.84418774 11.59600270
H 13.91644202 11.75248011 10.32142619

H 12.57649134 12.51307052 12.35271078

fe13s1e_0

Fe 9.91626890 9.87894074 10.00878834
Fe 11.73950401 9.96580777 8.47829038
Fe 12.12060626 10.23440667 10.91435710
Fe 7.66851003 9.61975535 9.22127349
Fe 8.13756183 9.91350935 11.64107733
Fe 8.47602856 11.75415973 9.93780267
Fe 10.90430328 11.94398837 9.52999484
Fe 8.90074851 7.85148557 10.57768127
Fe 11.41529009 8.07965303 10.20725402
Fe 10.38676021 8.91806064 12.20928100
Fe 10.12289589 11.30389729 11.86885018
Fe 9.81694456 8.50927358 8.12997041
Fe 9.35502599 10.82036169 7.83030187
S 7.82777177 7.41598047 8.64932845
C 8.25268781 10.51427082 6.19793446
C 9.38913719 9.60391828 6.14373622
H 8.26358038 11.40816286 5.54315710
H 7.24643775 10.07769987 6.33780828
H 10.22658796 9.85043872 5.46417074
H 9.14914632 8.50915825 6.11951366

fe13s1e_1

Fe 10.01030375 9.86750641 9.90397805
Fe 12.05598596 9.57902269 8.68695080
Fe 12.05360887 9.93561292 11.18371071
Fe 7.90885507 9.91000289 8.77597520
Fe 8.02603298 10.20946445 11.22847431
Fe 8.82142780 11.92064362 9.64697048
Fe 11.34684309 11.74125159 9.56239818
Fe 8.78010818 7.98615517 10.35768213
Fe 11.27452174 7.90923911 10.22481092
Fe 10.01648124 8.99580864 12.17900159
Fe 10.14030479 11.35647443 11.74992734
Fe 9.91935844 8.57983695 7.92938675
Fe 10.02109133 10.87738003 7.69931794
S 7.82753649 7.67098022 8.34448268
C 10.01060818 11.00732173 5.73612535
C 10.15395688 9.56045925 5.98191998
H 10.89217977 11.55104786 5.34428751
H 9.04798107 11.36620477 5.32525721
H 11.11658691 9.12053596 5.64878453
H 9.28628488 8.94763377 5.65050598

fe13s1e_2

Fe 9.93443861 10.10012505 10.10134901
Fe 12.00689733 10.35188832 8.83710155
Fe 11.92504698 10.06814782 11.38692554
Fe 7.84933090 10.04900199 8.91138176
Fe 7.86987823 9.87243534 11.36933562
Fe 8.51836755 11.97851023 10.18877565
Fe 11.16777709 12.09673406 10.06777313
Fe 8.86232736 8.00622707 10.10855584

Fe 11.38846582 8.23810524 10.00601219
Fe 9.95594541 8.73766839 12.11731330
Fe 9.84185663 11.17614701 12.14625696
Fe 10.06779446 9.06444614 8.01924898
Fe 9.75418716 11.46767412 8.13252539
S 8.04935009 7.98078181 7.98121356
C 12.98468317 12.36083083 9.00769280
C 13.63774936 11.26781937 8.24118603
H 13.56688101 12.68806634 9.90069034
H 12.68867571 13.23515134 8.38534257
H 14.56832040 10.83848123 8.65931142
H 13.66482716 11.37929767 7.13903136

Fe13S7A

Fe	9.97523788	10.04438146	9.70817994
Fe	11.84933893	10.08194830	8.34783206
Fe	12.15641774	9.97764781	10.90202473
Fe	8.06717259	10.04166254	8.41639784
Fe	7.96512969	10.02383363	11.09097404
Fe	8.84493943	12.24940816	9.79623258
Fe	11.40105161	12.07210399	9.85420884
Fe	8.68476345	7.99609686	10.01810691
Fe	11.19376704	8.00484969	9.87789913
Fe	10.19151457	8.74221829	11.92567486
Fe	9.99901322	11.21203176	11.77506375
Fe	9.99277267	8.42764439	7.74601928
Fe	10.03680528	11.74465791	7.80578630
S	7.87034050	7.77342787	7.96220860
C	9.22536718	10.16202806	6.80440639
C	10.64798206	10.15078256	6.74720393
H	8.60407389	10.14908128	5.87920240
H	11.20178899	10.13483279	5.78113830
S	8.10686752	8.12988944	12.24992434
S	12.27641797	7.94446329	11.82382182
S	6.79555466	11.39629888	9.79808194
S	10.29967076	13.70265061	8.80308549
S	12.19037262	11.95041166	11.91605662
S	12.15552636	7.82528948	7.92542829

Fe13S7E

Fe	10.02022632	9.91209332	9.82204268
Fe	12.12239523	10.04302258	8.79579361
Fe	12.11210706	10.04337921	11.23931389
Fe	7.82983910	9.97448677	8.73529457
Fe	7.93196358	10.09270309	11.15249394
Fe	8.89715842	12.00230797	9.51766583
Fe	11.30630578	12.08775534	9.79980755
Fe	8.72048916	7.83890131	9.99909504
Fe	11.30336490	7.87793643	9.89420624
Fe	10.12674464	8.62921074	11.87317194
Fe	10.01080830	11.26249753	11.70394066
Fe	9.98686709	8.50884838	7.87867243
Fe	10.02335905	10.94929250	7.65290683
S	7.84295254	7.86790270	7.92466374
S	8.00946728	8.11431444	12.15595676
S	12.24655326	7.92309364	11.92938695

S	6.80297048	11.60454532	9.99608445
S	10.25418956	13.11899253	8.18374502
S	12.15254626	12.18293593	11.81960465
S	12.17912741	7.97488014	7.84397705
C	9.83760050	11.22044387	5.66849757
C	10.04187253	9.81813022	5.87874627
H	10.66724711	11.84733329	5.29738028
H	8.82725637	11.61297169	5.45407850
H	11.02627523	9.40953492	5.58644904
H	9.19188026	9.14244098	5.65906248

Fe13S20A

Fe	10.07685300	10.00461203	9.88768081
Fe	12.26821451	9.97928106	8.45087751
Fe	12.37036713	9.98416163	11.22498017
Fe	7.81187867	10.02029146	8.56894816
Fe	7.86427493	10.02641661	11.34654762
Fe	8.71422020	12.25577181	9.93441863
Fe	11.47562547	12.22859592	9.86624910
Fe	8.66470323	7.75628924	9.93616259
Fe	11.45171849	7.72865840	9.86015390
Fe	10.13152251	8.63808113	12.10843930
Fe	10.15052308	11.40276438	12.14991628
Fe	10.00794429	8.60404825	7.66201439
Fe	10.02135086	11.70182938	7.11432088
S	7.87166151	7.86994729	7.84505680
S	7.98780594	7.89126514	12.09868651
S	12.27137284	7.84516853	11.97744656
S	6.63616282	11.34255644	10.00772115
S	10.07582136	13.45034536	8.57446230
S	12.29645187	12.10873304	11.99807446
S	12.14114814	7.82545791	7.72841153
S	10.13463095	13.44931554	11.23179777
S	8.01625574	12.15322241	12.11092884
S	8.85021410	10.01794469	13.39391249
S	11.50097253	9.99948393	13.31458992
S	10.07963679	6.54170821	11.22624154
S	13.54598732	11.28142151	9.81944221
S	12.18638894	12.11570054	7.74943614
S	7.88983073	12.14764847	7.85424203
S	6.60563501	8.69915050	10.00165932
S	10.00561068	6.51235616	8.59905914
S	13.52437312	8.64187611	9.81878409
S	11.30053496	9.94748737	6.40656672
S	8.67439815	9.97577580	6.46922166
C	9.39395044	12.75489248	5.43139763
C	10.64586263	12.66800569	5.36954654
H	8.39462597	13.03931450	5.10328606
H	11.64367712	12.80362242	4.95355472

Fe13S20E

Fe	10.04581475	9.95956704	9.97445217
Fe	12.15752538	9.93688716	8.47330708
Fe	12.37044583	9.99492011	11.29900297
Fe	7.84419997	9.94117886	8.60665117
Fe	7.81197681	10.00100717	11.44623866

Fe	8.73166901	12.29243893	10.10801361
Fe	11.36854353	12.28757283	10.02111972
Fe	8.65972626	7.71113918	10.02258218
Fe	11.43451549	7.70830679	9.93374232
Fe	10.12145954	8.57306537	12.20559624
Fe	10.11697405	11.27007641	12.22198864
Fe	9.96917539	8.50156325	7.62276776
Fe	9.94538641	11.22550900	6.87920288
S	7.86252031	7.80831974	7.92344395
S	7.96329092	7.86118074	12.16768179
S	12.27504482	7.85301507	12.02435618
S	6.71987056	11.34734263	9.98713085
S	10.02308128	13.94756860	9.17326690
S	12.21236384	12.10643681	12.10659656
S	12.09241712	7.80042087	7.79342439
S	10.09504412	13.51053316	11.31105111
S	8.01673454	12.11589136	12.24685479
S	8.82153000	9.94248885	13.47429601
S	11.49731463	9.94350743	13.38938007
S	10.09353026	6.50425503	11.30229458
S	13.37138598	11.34305453	9.77645403
S	11.72311376	12.04558928	7.81478683
S	8.22926780	12.04951515	7.92681941
S	6.62381790	8.68676792	10.05312931
S	10.00143869	6.49766024	8.65888357
S	13.47006743	8.68181545	9.83924148
S	11.41078569	9.70747419	6.29355538
S	8.43910057	9.72138119	6.37762459
C	9.88908006	13.02806234	5.79859869
C	9.94077597	11.92182069	4.92547793
H	10.79138452	13.64374296	5.96012429
H	8.94204275	13.57637531	5.94496600
H	10.88159667	11.67795692	4.40325844
H	9.03125930	11.60036830	4.38988811

Fe55S1A_0

Fe	13.71694008	12.26612215	12.48820121
Fe	15.66931505	12.29090798	11.29761962
Fe	15.64763281	12.28863710	13.69129906
Fe	11.77345086	12.25779421	11.24441420
Fe	11.73593169	12.26486978	13.64012781
Fe	12.44935381	14.19924889	12.43718273
Fe	14.88082017	14.22634560	12.48842153
Fe	12.52816158	10.30683078	12.47469338
Fe	14.93380008	10.33621485	12.50978382
Fe	13.70109416	11.07925888	14.43418471
Fe	13.68525913	13.48155099	14.42713318
Fe	13.76519116	11.04971921	10.55041871
Fe	13.71678422	13.46136090	10.52925169
Fe	17.74162624	12.34092217	10.03740041
Fe	17.75339329	12.30968801	12.49692766
Fe	17.71083350	12.29856724	14.96035501
Fe	9.97658119	12.01696319	9.74952331
Fe	9.57924228	12.36598104	12.40177356
Fe	9.71247356	12.27245431	14.96726934
Fe	11.27458258	16.22540720	12.46458860
Fe	13.72612878	16.28533625	12.45897125

Fe 16.17465359 16.30936717 12.49278135
Fe 11.23956017 8.26119588 12.53591189
Fe 13.72493925 8.23495700 12.52970974
Fe 16.20396946 8.28158027 12.56523514
Fe 13.70176855 9.80076080 16.50211763
Fe 13.73026479 12.25907038 16.52563554
Fe 13.69586957 14.74073202 16.49839383
Fe 13.92043188 9.70180818 8.49612044
Fe 13.80494094 12.25206186 8.46251768
Fe 13.72811228 14.77556122 8.49609947
Fe 15.71936826 11.03827182 15.73328560
Fe 15.70616737 13.53034060 15.74091792
Fe 15.79902022 11.04371277 9.25448693
Fe 15.74545677 13.54924535 9.23496189
Fe 11.70019208 11.01682111 15.71921741
Fe 11.71666365 13.47388323 15.78935311
Fe 11.93225517 10.87255462 9.13541732
Fe 11.75935157 13.43671742 9.17129788
Fe 16.93893863 14.32873538 11.25293135
Fe 16.94384945 14.30551083 13.72702841
Fe 10.23393327 14.25008914 11.10964709
Fe 10.51293532 14.26059101 13.85776516
Fe 16.98684338 10.29182685 11.27810671
Fe 16.99088985 10.27719629 13.75764014
Fe 10.53002154 10.22624557 11.20386897
Fe 10.41892686 10.29438447 13.67746470
Fe 12.50932892 15.54395096 14.49805895
Fe 14.95256544 15.54626862 14.49837479
Fe 12.43686312 9.03084940 14.51146632
Fe 14.94067260 9.02354512 14.52809537
Fe 12.39596502 15.49158839 10.44663109
Fe 14.91174491 15.55175275 10.47777820
Fe 12.53945513 8.96120963 10.49440743
Fe 15.02089539 8.99092918 10.52567108
C 9.45537930 15.57301423 12.59144071
C 8.87327998 14.29339051 12.79523652
H 8.78951205 16.47277417 12.59754648
H 7.77355787 14.19132034 12.90010102
S 8.26277402 13.00878488 10.48622347

Fe55S1A_1

Fe 13.71324285 12.27643078 12.48548484
Fe 15.66256313 12.27484349 11.29566722
Fe 15.64650846 12.30917496 13.69924026
Fe 11.76504873 12.32098906 11.27559540
Fe 11.77392167 12.26206237 13.69704935
Fe 12.47418301 14.23772187 12.47062204
Fe 14.89891809 14.23939312 12.48864069
Fe 12.50470666 10.33101612 12.47678863
Fe 14.91190031 10.34719227 12.50316389
Fe 13.72137937 11.09264167 14.43741758
Fe 13.67771136 13.51108957 14.41870622
Fe 13.71376372 11.06230667 10.54466921
Fe 13.74761132 13.48773076 10.54645652
Fe 17.72601499 12.22298382 10.02956332
Fe 17.74018088 12.26739988 12.51786870
Fe 17.71377921 12.34913853 14.98171852

Fe 9.76381221 12.29089982 9.92284121
Fe 9.74492755 12.30728658 12.52344846
Fe 9.74596993 12.11981980 15.00111579
Fe 11.13305743 16.21602348 12.35404546
Fe 13.71949922 16.31544566 12.51885147
Fe 16.19033993 16.30201796 12.51144686
Fe 11.23934456 8.26750461 12.44496419
Fe 13.70191489 8.26074347 12.48707769
Fe 16.18383047 8.28928193 12.52325577
Fe 13.77524988 9.82009084 16.50732272
Fe 13.72720612 12.29636927 16.51280857
Fe 13.61252044 14.81575390 16.45178895
Fe 13.70429468 9.78161212 8.48885951
Fe 13.70720789 12.28111365 8.47282138
Fe 13.86758353 14.75731210 8.47969765
Fe 15.74280862 11.08055358 15.75040441
Fe 15.68484196 13.57090017 15.73777348
Fe 15.71423206 11.00917742 9.24312745
Fe 15.77687153 13.48398228 9.23323810
Fe 11.73988858 10.98641826 15.75433645
Fe 11.69222733 13.48425073 15.78658747
Fe 11.73851124 11.08642584 9.21669044
Fe 11.75620915 13.63242320 9.25521698
Fe 17.01259723 14.25038020 11.28772363
Fe 16.95435720 14.32179125 13.74876069
Fe 10.31157320 14.28496735 11.16152511
Fe 10.49252470 14.26045501 13.86262287
Fe 16.96177148 10.24342493 11.25497403
Fe 16.96218400 10.29461259 13.75242089
Fe 10.46315740 10.29705999 11.24059099
Fe 10.45480451 10.20534692 13.71267883
Fe 12.41415672 15.57374612 14.47223948
Fe 14.92430992 15.57084164 14.50816517
Fe 12.49232817 9.03073258 14.49503013
Fe 14.96533787 9.02868778 14.51784186
Fe 12.71695372 15.56665509 10.50074590
Fe 15.10457279 15.54225313 10.48485199
Fe 12.43501298 9.02395345 10.44385359
Fe 14.93095081 9.00671498 10.49723616
C 9.32594942 15.39014658 12.66606333
C 8.83341556 14.05714357 12.58008515
H 8.61087546 16.24588644 12.65823710
H 7.73216004 13.87274394 12.59186879
S 10.70925793 16.19892232 10.03019664

Fe55S1A_2

Fe 13.71170028 12.26975749 12.49688649
Fe 15.65245572 12.28940319 11.29414911
Fe 15.65184954 12.29532207 13.69246812
Fe 11.77164179 12.28516404 11.27381280
Fe 11.76416701 12.26659344 13.70917656
Fe 12.47462422 14.21594965 12.48172218
Fe 14.88876043 14.23673988 12.49536431
Fe 12.50532697 10.32418913 12.48874804
Fe 14.91724662 10.34115142 12.50012599
Fe 13.71988679 11.07969284 14.43820648
Fe 13.69505304 13.48980959 14.43677353

Fe 13.72166890 11.06572349 10.54474833
Fe 13.71309263 13.47753186 10.54817569
Fe 17.71709201 12.31120724 10.00859649
Fe 17.73670007 12.30108713 12.48694452
Fe 17.72463545 12.31123548 14.95508907
Fe 9.80674983 12.15004398 9.91067930
Fe 9.73564405 12.33581874 12.48450145
Fe 9.70860247 12.22999910 14.99574536
Fe 11.25668747 16.23585189 12.50692210
Fe 13.73782886 16.30445967 12.51150523
Fe 16.18113107 16.30909603 12.48963746
Fe 11.22051303 8.27020158 12.51376459
Fe 13.70424110 8.25549380 12.51433229
Fe 16.19335060 8.28172568 12.50833894
Fe 13.74560423 9.79880581 16.50011049
Fe 13.72470523 12.27215061 16.52582527
Fe 13.69513510 14.75035312 16.51181641
Fe 13.78303625 9.76734919 8.49323820
Fe 13.76502253 12.24809291 8.46009231
Fe 13.64122712 14.78439440 8.53183918
Fe 15.73781382 11.05544354 15.73902144
Fe 15.71400823 13.53620623 15.74557030
Fe 15.76575672 11.02932568 9.25348384
Fe 15.70770085 13.53068017 9.24484134
Fe 11.72280126 11.02204165 15.75031374
Fe 11.70308363 13.50268461 15.78045120
Fe 11.78123819 10.97577334 9.19654968
Fe 11.75221320 13.44289323 9.20680303
Fe 16.95531802 14.30389455 11.24686947
Fe 16.95164940 14.31417896 13.73213947
Fe 10.40667688 14.28915494 11.07982558
Fe 10.45494529 14.25311899 13.80765444
Fe 16.97003900 10.27784634 11.26238736
Fe 16.97432605 10.28331647 13.74269074
Fe 10.47394765 10.25691361 11.25880650
Fe 10.44095219 10.25967531 13.72809895
Fe 12.46764781 15.53112148 14.52579480
Fe 14.94752752 15.54974682 14.52050897
Fe 12.46233570 9.03079763 14.51743186
Fe 14.96190911 9.02109597 14.51098761
Fe 12.51698533 15.53894625 10.52659205
Fe 14.95931970 15.55965001 10.49696487
Fe 12.46858487 8.99574038 10.48506901
Fe 14.95820693 9.00531525 10.50466273
C 9.42611830 15.46149555 12.47703849
C 8.89098703 14.13674053 12.46867648
H 8.72853103 16.33293041 12.43186267
H 7.78561394 13.98486346 12.45457209
S 10.78359112 15.42547865 9.17041154

Fe55S1A_3

Fe 13.70933897 12.27352994 12.49632522
Fe 15.65353946 12.29491735 11.29550817
Fe 15.65931077 12.29610733 13.69121103
Fe 11.79100062 12.28430528 11.26020028
Fe 11.76200444 12.27695353 13.70571236
Fe 12.49551499 14.22446432 12.47402024

Fe 14.89037203 14.23517504 12.49642769
Fe 12.50698969 10.32801838 12.49056446
Fe 14.92554321 10.34190907 12.50014347
Fe 13.72500176 11.07962631 14.43490921
Fe 13.69875747 13.48550368 14.43839207
Fe 13.73075429 11.06252819 10.55226621
Fe 13.71553298 13.48513126 10.52933943
Fe 17.73461506 12.31147692 10.02150729
Fe 17.74547808 12.31151414 12.48774256
Fe 17.72972155 12.31584420 14.95663345
Fe 9.78118617 12.17920059 9.89502753
Fe 9.74160521 12.37748500 12.44828480
Fe 9.72971281 12.24574406 15.00333358
Fe 11.25296803 16.25996470 12.48692811
Fe 13.73226384 16.29866962 12.52422711
Fe 16.17801914 16.31315954 12.52829616
Fe 11.20137782 8.29419506 12.51348687
Fe 13.70779045 8.26287565 12.52243940
Fe 16.19944078 8.28122779 12.51188534
Fe 13.75214726 9.79238331 16.48222626
Fe 13.73320186 12.26072944 16.52952577
Fe 13.68386896 14.72837730 16.52021552
Fe 13.78199274 9.72895984 8.51986241
Fe 13.78414462 12.22228782 8.45851179
Fe 13.77463414 14.71034593 8.47408209
Fe 15.75004676 11.04939368 15.73694019
Fe 15.71650943 13.53096464 15.74684600
Fe 15.76031851 11.02595046 9.26277513
Fe 15.75764638 13.51874108 9.24323500
Fe 11.72229007 11.01684473 15.74483061
Fe 11.70239424 13.49892437 15.78854318
Fe 11.76785712 10.94758820 9.22925936
Fe 11.71751583 13.40202689 9.14844932
Fe 16.95363069 14.31688324 11.26030449
Fe 16.95512462 14.31829737 13.74000607
Fe 10.47628446 14.29595584 11.08812013
Fe 10.45621744 14.28373153 13.81223324
Fe 16.97711632 10.28203538 11.25879457
Fe 16.98204993 10.28792891 13.73548066
Fe 10.46154587 10.27024964 11.25147049
Fe 10.42576801 10.28368861 13.71699582
Fe 12.46743208 15.53010697 14.52492471
Fe 14.94697286 15.54337551 14.53395104
Fe 12.45421100 9.03035477 14.51418587
Fe 14.96942617 9.01442657 14.50928054
Fe 12.51381551 15.59192315 10.49747146
Fe 14.97243422 15.54714947 10.49054679
Fe 12.48183205 8.97597280 10.50231249
Fe 14.97511050 8.99557511 10.51002905
C 9.42761257 15.48795257 12.46688138
C 8.89045955 14.16483374 12.44246082
H 8.71636484 16.34911343 12.45453018
H 7.78308990 14.02710025 12.43222230
S 11.66895875 15.51212905 8.42356586

Fe55S1E_0

Fe 12.49123870 13.63749912 12.54835101

Fe 14.43731264 13.55525000 11.37330739
Fe 14.40590136 13.61560260 13.78304614
Fe 10.54233543 13.63630591 11.32918224
Fe 10.51923276 13.72224819 13.73656405
Fe 11.31352687 15.59439383 12.49188234
Fe 13.72545050 15.54625801 12.49730290
Fe 11.22175031 11.71065838 12.58540639
Fe 13.63205375 11.64554429 12.60392379
Fe 12.39545553 12.49442421 14.53014762
Fe 12.50346767 14.90605889 14.45946746
Fe 12.47155061 12.38824655 10.63256177
Fe 12.52065972 14.78735988 10.57527748
Fe 16.52113228 13.44230743 10.16367752
Fe 16.50021879 13.48804394 12.62456140
Fe 16.45029050 13.56085115 15.06985339
Fe 8.48560892 13.66095045 10.03497241
Fe 8.43734278 13.78521430 12.46867997
Fe 8.50644912 13.72132412 15.00883904
Fe 10.04925562 17.66735757 12.45965964
Fe 12.56995791 17.64543109 12.42471785
Fe 15.05588541 17.57467869 12.41840948
Fe 9.98592029 9.69958409 12.59473349
Fe 12.36076850 9.57026234 12.61054014
Fe 14.80604417 9.52844491 12.71012460
Fe 12.40870500 11.31610443 16.65254608
Fe 12.53448338 13.82748177 16.58000441
Fe 12.54459478 16.29468971 16.45177707
Fe 12.51435491 11.09029453 8.57997271
Fe 12.54794266 13.55858155 8.51737052
Fe 12.55334461 16.01697120 8.48044696
Fe 14.42139700 12.42695604 15.85900206
Fe 14.54839011 14.96265497 15.76962432
Fe 14.52230374 12.26535581 9.35277108
Fe 14.55826103 14.74684770 9.29688458
Fe 10.51687749 12.63003084 15.92017334
Fe 10.50207395 15.07740700 15.76294805
Fe 10.50770962 12.39438253 9.24582899
Fe 10.52123947 14.85118147 9.25147688
Fe 15.81264570 15.51639820 11.28692355
Fe 15.78213960 15.58427074 13.73774450
Fe 9.27831878 15.69923888 11.22740963
Fe 9.24866288 15.74650343 13.74766640
Fe 15.70651753 11.48542158 11.42973504
Fe 15.61397962 11.52078500 13.92252975
Fe 9.20555068 11.66347668 11.19893884
Fe 9.08950908 11.79009243 13.59304343
Fe 11.30644268 16.99653352 14.44347711
Fe 13.80960886 16.93380063 14.45048745
Fe 10.88799345 10.54576202 14.71004947
Fe 13.46456069 10.39548541 14.63486218
Fe 11.32440491 16.86762494 10.45423043
Fe 13.82450115 16.80430000 10.44470311
Fe 11.20689121 10.37443792 10.55526320
Fe 13.68787268 10.30853049 10.63358015
C 10.98149600 7.98825324 13.12184073
C 10.20417508 8.51549263 14.24656791
H 10.60247835 7.12649882 12.53580984
H 12.09631772 7.83470760 13.34817991

H 9.21910291 8.07281733 14.50031130
H 10.81098657 8.68570354 15.20073788
S 8.61894200 11.48088928 15.74196876

Fe55S1+E_1

Fe 12.49874255 13.67020499 12.55838079
Fe 14.43574637 13.55487866 11.36147914
Fe 14.43768464 13.63962667 13.76667655
Fe 10.55183323 13.65629078 11.36245835
Fe 10.54413632 13.78167427 13.75842599
Fe 11.34653674 15.64374501 12.47975347
Fe 13.75568378 15.56701551 12.48886963
Fe 11.23723392 11.74251250 12.67627967
Fe 13.60696143 11.67342643 12.64951481
Fe 12.43489021 12.53787165 14.57074797
Fe 12.53447994 14.94610006 14.45121824
Fe 12.45532021 12.39406200 10.65342007
Fe 12.52532774 14.79498118 10.57762813
Fe 16.49905988 13.43632595 10.10043548
Fe 16.52266481 13.51167661 12.56167764
Fe 16.52054618 13.58898529 15.00769657
Fe 8.50269134 13.62824101 10.05751695
Fe 8.45580190 13.78981618 12.53093558
Fe 8.45415537 13.94266916 14.98735364
Fe 10.12160681 17.73277973 12.39017836
Fe 12.61652804 17.67897553 12.41072295
Fe 15.10379492 17.57613858 12.40546782
Fe 9.94340506 9.78966265 12.77775255
Fe 12.33111480 9.57911152 12.69706891
Fe 14.79954646 9.55086926 12.71047464
Fe 12.39644008 11.54189404 16.73184188
Fe 12.51086314 13.94437586 16.58826932
Fe 12.60060185 16.36064416 16.44397002
Fe 12.45324915 11.09316461 8.61128318
Fe 12.50781366 13.53892298 8.52795643
Fe 12.55421490 16.01189336 8.48927962
Fe 14.46018108 12.50936846 15.86631241
Fe 14.57396409 14.97723150 15.74293456
Fe 14.47786760 12.25295906 9.34082289
Fe 14.54528009 14.74006145 9.27867117
Fe 10.42727086 12.68890494 15.88541438
Fe 10.51637086 15.13895077 15.74623703
Fe 10.45508221 12.35119404 9.32448681
Fe 10.51754572 14.83016948 9.26382831
Fe 15.81680909 15.51559394 11.25021281
Fe 15.83398528 15.59790868 13.71600839
Fe 9.29945504 15.69883126 11.22772819
Fe 9.28215082 15.84455933 13.70841742
Fe 15.66095872 11.49402291 11.40179230
Fe 15.66607714 11.57023172 13.88162037
Fe 9.19160464 11.69653544 11.39619378
Fe 9.16091692 11.84949580 13.88940006
Fe 11.35774291 17.04338371 14.43062614
Fe 13.86653030 16.95841653 14.44411916
Fe 11.01935369 10.47388958 14.81642830
Fe 13.61722901 10.41604065 14.68779579
Fe 11.33680871 16.88970543 10.42796024

Fe 13.83873356 16.81571384 10.43287095
Fe 11.16287914 10.40804582 10.66333410
Fe 13.62521003 10.30188992 10.66816364
C 10.87117185 7.99713139 13.15813493
C 9.93641117 8.40592068 14.21183729
H 10.57092418 7.21333496 12.43499361
H 11.93628047 7.81665019 13.51491827
H 8.91252632 7.97665544 14.22797915
H 10.34746079 8.50675281 15.24728331
S 12.49614181 9.37775477 16.32891974

Fe55S1+E_2

Fe 12.48080182 13.66306685 12.55151359
Fe 14.43248924 13.55036643 11.37024146
Fe 14.41595089 13.61008076 13.77111518
Fe 10.54010736 13.64452207 11.33249295
Fe 10.52102345 13.75875235 13.74234508
Fe 11.33647020 15.64375006 12.47528288
Fe 13.74170124 15.54157949 12.49614928
Fe 11.17134476 11.71169223 12.62475028
Fe 13.57725009 11.65992025 12.61758365
Fe 12.41130647 12.52495030 14.53938051
Fe 12.51219806 14.92471126 14.45215430
Fe 12.45894924 12.39379421 10.63734974
Fe 12.51594137 14.79269834 10.57261987
Fe 16.51747607 13.45438593 10.14292261
Fe 16.51009103 13.48217960 12.59588396
Fe 16.46395706 13.54245603 15.05412722
Fe 8.49868560 13.60496524 9.97049889
Fe 8.47687683 13.76183575 12.50300707
Fe 8.46200955 13.88690938 15.05899611
Fe 10.10223408 17.72216514 12.40505882
Fe 12.59911967 17.67456675 12.41034506
Fe 15.08366127 17.53759481 12.43351225
Fe 9.88884555 9.80474449 12.74796765
Fe 12.32653873 9.62062367 12.64113766
Fe 14.76180397 9.50317929 12.69521977
Fe 12.35452135 11.28361332 16.64520114
Fe 12.50103785 13.80339901 16.57588817
Fe 12.58279277 16.28368971 16.45689733
Fe 12.52016727 11.08905316 8.58733523
Fe 12.54315470 13.55199231 8.51218978
Fe 12.53840967 16.02911455 8.49307801
Fe 14.41113189 12.41706076 15.85267970
Fe 14.53826889 14.92576248 15.76887415
Fe 14.50637248 12.26599235 9.35383149
Fe 14.55007026 14.75136509 9.29605264
Fe 10.43259592 12.65532266 15.87917779
Fe 10.51668537 15.12348115 15.73553724
Fe 10.49476882 12.36932477 9.26937631
Fe 10.52022548 14.84102273 9.25262142
Fe 15.83058978 15.51109979 11.26782140
Fe 15.81060059 15.56194934 13.75309475
Fe 9.28913173 15.67176006 11.22619276
Fe 9.27446541 15.79355429 13.69405668
Fe 15.64405978 11.48199999 11.41764645
Fe 15.60173580 11.52627582 13.87952294

Fe 9.17893411 11.70077331 11.26624645
Fe 9.09493683 11.87438432 13.92784549
Fe 11.34709879 17.03560414 14.42570531
Fe 13.84102497 16.93518497 14.46101022
Fe 11.03396720 10.58048413 14.72338465
Fe 13.51789693 10.40510186 14.65239730
Fe 11.30976709 16.89431809 10.42809564
Fe 13.82104842 16.80407735 10.44859233
Fe 11.15679148 10.42943802 10.59833693
Fe 13.61872080 10.29215225 10.65015221
C 10.89164274 8.10266352 13.21312509
C 10.16911050 8.62048558 14.38733656
H 10.48672996 7.24029540 12.64532664
H 12.01678113 7.93132449 13.39739574
H 9.20960525 8.17110014 14.70615919
H 10.83254788 8.79216888 15.31041000
S 7.79445631 10.62519092 12.61707229

Fe55S1+E_3

Fe 12.47933077 13.67194607 12.55090948
Fe 14.43053968 13.55175958 11.37485217
Fe 14.40010703 13.61512611 13.77915838
Fe 10.53654287 13.66648215 11.33677637
Fe 10.53405472 13.79267593 13.75320244
Fe 11.33411661 15.64374780 12.46392215
Fe 13.75318671 15.55485018 12.49260853
Fe 11.21587679 11.74527643 12.64837322
Fe 13.58527826 11.66733054 12.62545707
Fe 12.38175790 12.54109715 14.56379176
Fe 12.51785970 14.95719938 14.44961112
Fe 12.44648262 12.39963237 10.64873079
Fe 12.52931474 14.79433113 10.57201849
Fe 16.51610354 13.43949224 10.14373903
Fe 16.49168900 13.46822690 12.60842932
Fe 16.47091070 13.52039636 15.05284546
Fe 8.48466835 13.61944774 10.05002653
Fe 8.46562718 13.79279408 12.53296406
Fe 8.46649395 13.99274532 15.04233958
Fe 10.10326250 17.71969880 12.35292543
Fe 12.61797271 17.66823545 12.38718762
Fe 15.10271409 17.56333627 12.42097493
Fe 9.93847519 9.76518542 12.72650420
Fe 12.30740956 9.58925522 12.63477398
Fe 14.75146952 9.54230013 12.67074864
Fe 12.38462716 11.45282415 16.67046635
Fe 12.48802334 13.88678444 16.58676609
Fe 12.61922430 16.34993540 16.43012323
Fe 12.45867409 11.11632398 8.59687448
Fe 12.52066193 13.55000052 8.52015786
Fe 12.57962919 16.00789973 8.48398205
Fe 14.43172194 12.46109079 15.86076657
Fe 14.54552249 14.94334469 15.76171718
Fe 14.49024216 12.27448645 9.35407798
Fe 14.56778055 14.74726089 9.29608667
Fe 10.34969983 12.76627645 15.90395233
Fe 10.52093662 15.20494325 15.72784597
Fe 10.44643386 12.37186978 9.30476043

Fe 10.51922660 14.83256888 9.24652648
Fe 15.83241011 15.50167922 11.28942490
Fe 15.80524201 15.55955871 13.75522152
Fe 9.27545689 15.69838449 11.19381010
Fe 9.28195086 15.85272127 13.68981991
Fe 15.65887350 11.48202532 11.40920570
Fe 15.60292694 11.51080823 13.90569198
Fe 9.17325577 11.69966361 11.37931075
Fe 9.14323581 11.87461647 13.86104510
Fe 11.36820083 17.07865777 14.39314104
Fe 13.86627723 16.96119711 14.44205685
Fe 10.94354665 10.48456883 14.75954419
Fe 13.47462755 10.43590133 14.66131428
Fe 11.33721025 16.87959970 10.40650629
Fe 13.85203162 16.80969118 10.43927735
Fe 11.13431529 10.41447719 10.62222342
Fe 13.62569947 10.31100808 10.63141626
C 10.89382714 7.99928942 13.09786085
C 10.15381109 8.46006402 14.27588823
H 10.48157839 7.19857314 12.45194465
H 12.00628687 7.80474852 13.28389491
H 9.16174852 8.02755000 14.51958916
H 10.75880800 8.56605002 15.22403652
S 10.28031023 10.78478969 16.93803337

Fe55S5+A

Fe 13.74730875 12.26099713 12.47484707
Fe 15.67801919 12.28161247 11.30897711
Fe 15.65421196 12.28110102 13.70287791
Fe 11.71349028 12.31325484 11.30153514
Fe 11.70880273 12.21588117 13.64861267
Fe 12.45452375 14.24021629 12.43925801
Fe 14.93069096 14.24325363 12.481059
Fe 12.54372352 10.312138 12.45801699
Fe 14.92782714 10.3516002 12.48726277
Fe 13.71090653 11.09181996 14.44784486
Fe 13.65577905 13.50945141 14.39159356
Fe 13.7472439 11.07269426 10.52465251
Fe 13.75193086 13.47910034 10.53767156
Fe 17.76413404 12.2640302 10.05291545
Fe 17.76275252 12.24234812 12.53944432
Fe 17.7171699 12.28651088 14.98661793
Fe 9.9581904 12.14171772 9.80390932
Fe 9.46984704 12.20322861 12.4319324
Fe 9.77846436 12.16717484 14.93874139
Fe 11.26251703 16.23048572 12.20876153
Fe 13.7683643 16.31338513 12.49137311
Fe 16.17357255 16.34220427 12.51870357
Fe 11.29776331 8.23232407 12.4868528
Fe 13.75673281 8.2323593 12.47613883
Fe 16.1892954 8.27486932 12.49042611
Fe 13.79658725 9.85777795 16.51983317
Fe 13.73275254 12.31836274 16.47386522
Fe 13.63761817 14.84157318 16.41759349
Fe 13.8012631 9.79363588 8.44106367
Fe 13.7171894 12.31622194 8.45076393
Fe 13.84500722 14.71525356 8.447548

```
Fe  15.78368565  11.04506064  15.7940816
Fe  15.67125869  13.52945855  15.70930001
Fe  15.74747535  11.06301507  9.23011068
Fe  15.80268588  13.50457784  9.25831313
Fe  11.74752421  10.94083349  15.74486557
Fe  11.74637212  13.36280637  15.68802444
Fe  11.87639924  11.02882028  9.17447833
Fe  11.68722869  13.70022981  9.3481732
Fe  17.0658807  14.24778956  11.3449814
Fe  16.94712097  14.30428243  13.79087533
Fe  9.98299693  14.24502103  11.3008841
Fe  10.47568355  14.22089134  13.764434
Fe  16.98491727  10.26526133  11.24414292
Fe  16.97593538  10.25700785  13.75109969
Fe  10.58119286  10.22155784  11.17567964
Fe  10.47672379  10.17257634  13.69768064
Fe  12.36500661  15.55332614  14.53125211
Fe  14.91314278  15.54953451  14.48507726
Fe  12.51934191  8.99763788  14.49010253
Fe  14.9807231  9.04604637  14.50950181
Fe  12.86517165  15.58236952  10.46123024
Fe  15.19533187  15.52536105  10.47469552
Fe  12.53414171  8.97520824  10.42649421
Fe  15.00550848  9.01756598  10.45792783
S  8.18334152  12.94963998  10.66233868
S  10.7613213  15.79806694  9.84601564
S  12.38206212  17.59424478  13.61715288
S  10.554414  15.14677579  15.78754854
S  8.35241079  10.8344083  13.83690472
C  9.39262515  15.60829853  12.67889009
C  8.76786899  14.37802495  12.90001187
H  8.86650445  16.58852035  12.75192211
H  7.69614855  14.18765469  13.07875017
```

Fe55S5+E

```
Fe  13.69041473  12.24821765  12.44195071
Fe  15.66856061  12.30228959  11.36579987
Fe  15.54580607  12.29685237  13.76116543
Fe  11.76569799  12.22948071  11.15385672
Fe  11.62114582  12.2229186  13.5644717
Fe  12.41075715  14.20992113  12.39146607
Fe  14.82641008  14.23438339  12.51363768
Fe  12.52930657  10.28937243  12.39109498
Fe  14.90967122  10.35284681  12.52441652
Fe  13.56903684  11.06333118  14.40190576
Fe  13.5039886  13.465095  14.39450667
Fe  13.81089301  11.05389407  10.49852656
Fe  13.74969543  13.47162278  10.50743328
Fe  17.78366753  12.31555717  10.18827349
Fe  17.69532954  12.34261079  12.68072533
Fe  17.56743714  12.32838891  15.13548227
Fe  9.84286946  12.12313776  9.72526668
Fe  9.59401299  12.12412805  12.18868091
Fe  9.44132568  12.16586913  14.53143678
Fe  11.22195358  16.24073797  12.26962065
Fe  13.64248062  16.26547181  12.41424368
Fe  16.03451951  16.35883613  12.52833705
```

Fe 11.36132135 8.14933226 12.38297932
Fe 13.78535573 8.24237407 12.46170742
Fe 16.2268514 8.33013261 12.59741989
Fe 13.45325572 9.78409319 16.47382797
Fe 13.43596028 12.25338595 16.46581399
Fe 13.52950545 14.64798182 16.51051332
Fe 13.92951378 9.78466345 8.43677174
Fe 13.86906383 12.28795648 8.43997969
Fe 13.8713651 14.8329652 8.48497547
Fe 15.51024868 11.06047028 15.81170617
Fe 15.52074578 13.50799408 15.77860743
Fe 15.86753513 11.04174716 9.2907809
Fe 15.8165691 13.520239 9.31142477
Fe 11.49306561 10.98002811 15.5931272
Fe 11.39991892 13.4506969 15.63025627
Fe 11.9031381 10.96677175 9.09196707
Fe 11.84207864 13.4809138 9.09836382
Fe 16.9542063 14.31860001 11.39962015
Fe 16.77738776 14.34818378 13.86393808
Fe 10.41623395 14.21871143 10.9636085
Fe 10.35297473 14.19235806 13.49770047
Fe 17.02594622 10.3166522 11.38655236
Fe 16.91699118 10.32686186 13.89055102
Fe 10.59878411 10.16058457 11.04349027
Fe 10.52032061 10.11687671 13.56998406
Fe 12.18413586 15.51590371 14.49224246
Fe 14.703137 15.50862224 14.52423396
Fe 12.4267992 8.94943853 14.43766343
Fe 14.89512818 9.05123734 14.5550714
Fe 12.57233162 15.5192089 10.38037256
Fe 15.02948419 15.55734453 10.49114493
Fe 12.61396685 8.96654113 10.37798375
Fe 15.09977205 9.0190391 10.49702189
S 8.37148049 13.40316236 10.88491692
S 10.51417006 16.29713183 10.15451685
S 12.37554561 17.56783275 13.6596556
S 11.50314685 15.47826614 16.65230089
S 8.42342035 10.56725491 13.32516143
C 7.70628263 13.13277849 14.80249548
C 8.78874287 14.0519993 15.05496036
H 7.10273259 13.23065205 13.88342285
H 7.16842872 12.69582663 15.66599082
H 8.84216769 14.96555589 14.36151139
H 8.98991797 14.3838652 16.09253608

References

[1] B. Orbán and T. Höltzl, “The promoter role of sulfur in carbon nanotube growth,” *Dalt. Trans.*, vol. 51, no. 24, pp. 9256–9264, 2022, https://doi.org/10.1039/D2DT00355D
